# *Argopistes sexvittatus* and *Argopistes capensis* (Chrysomelidae: Alticini): Mitogenomics and Phylogeny of Two Flea Beetles Affecting Olive Trees

**DOI:** 10.3390/genes13122195

**Published:** 2022-11-23

**Authors:** Vaylen Hlaka, Maurizio Biondi, Elleunorah Allsopp, Barbara van Asch

**Affiliations:** 1 Department of Genetics, University of Stellenbosch, Stellenbosch 7602, South Africa; 2 Department of Life, Health and Environmental Sciences, University of L’Aquila, 67100 L’Aquila, Italy; 3 Agricultural Research Council, Infruitec-Nietvoorbij, Stellenbosch 7600, South Africa

**Keywords:** DNA barcodes, mitochondrial, *Olea europaea*, olive flea beetles, phylogenetics, South Africa

## Abstract

The genus *Argopistes* (Chrysomelidae: Alticini) is the only group of flea beetles specialized in plant hosts in the family Oleaceae. In southern Africa, *Argopistes* are often found feeding on African Wild Olive (*Olea europaea* subsp. *cuspidata*) and European cultivated olive (*O. e.* subsp. *europaea*), and heavy infestations can be devastating to mature trees and compromise the development of young trees. Despite their negative agricultural impact, African *Argopistes* are an understudied group for which no genetic data were available. We assessed the species diversity of olive flea beetles in the Western Cape province of South Africa, the largest olive-producing region in sub-Saharan Africa, by collecting adult specimens on wild and cultivated olive trees between 2015 and 2017. *Argopistes sexvittatus* Bryant, 1922 (*n* = 289) dominated at all sampling sites, and *Argopistes capensis* Bryant, 1944 (*n* = 2) was found only once. *Argopistes oleae* Bryant, 1922, a third species previously reported in the region, was not found. The complete mitogenomes of one *A. capensis* and two *A. sexvittatus* (striped and black morphotypes) individuals were sequenced for phylogenetic reconstruction in the context of other 64 species. The two olive flea beetle species form a monophyletic clade with other *Argopistes*, supporting the hypothesis that the exclusive feeding habit on Oleaceae is an evolutionary adaptation in this genus.

## 1. Introduction

Flea beetles (Chrysomelidae: Alticini) are small to moderately sized jumping beetles characterized by enlarged hindleg femora that allow the insect to spring into action when disturbed. The tribe Alticini is closely related to the tribe Galerucini, both contained in the subfamily Galerucinae [1]. Alticini includes 4000 to 8000 species in approximately 500 genera with worldwide distribution but occurring mainly in the tropical regions of South America, Africa and Asia [2]. Most flea beetles are highly specialized feeders, and adults bore small holes on the external surface of leaves, stems, and, more rarely, flowers of a wide range of higher plants. Larvae show different feeding habits, mainly root feeders, leaf miners, and external leaf feeders. Pupation generally occurs in the soil. Adult flea beetle feeding exposes the lower epidermis of the leaves to high levels of sunlight, eventually causing defoliation [3]. Leaf damage also leads to a loss of vigour of surrounding tissues, aggravated in hot and dry conditions. Heavy flea beetle infestations can be detrimental at the initial stages of plant development and may lead to the death of the host if not addressed. Flea beetles affect numerous agricultural and ornamental plants of economic significance. For example, the crucifer flea beetle *Phyllotreta cruciferae* (Goeze), the striped flea beetle *Phyllotreta striolata* (Fabricius), Zimmermann’s flea beetle *Phyllotreta zimmermanni* (Crotch) and the pale-striped flea beetle *Systena blanda* Melsheimer feed on cruciferous crops [3], *Epitrix fuscula* Crotch feeds on eggplants, and *Epitrix hirtipennis* (Melsheimer) defoliates tobacco. Three *Chaetocnema* species, *C. aridula* (Gyllenhal), *C. hortensis* Geoffroy and *C. mannerheimi* (Gyllenhal), are pests of cereals, and *C. concinna* (Marsham) is a pest of beetroot, buckwheat, sorrel, and strawberry [4]. *Luperomorpha xanthodera* (Fairmaire) is a known pest of ornamental plants in floriculture, especially roses [5].

Olive flea beetles are found in southern Africa, feeding on the leaves and stems of African Wild Olive (*O. e.* subsp. *cuspidata*) and European cultivated olive trees (*O. e.* subsp. *europaea*), and some species occasionally feed on olive fruits and may become serious pests [6]. In total, 15 species of *Argopistes* have been associated with *O. europaea* and other Oleaceae, and the genus appears to be specific to hosts in the olive family [2]. Bryant (1922) described and illustrated adults of *A. oleae* and *A. sexvittatus* [7] and later described *A. capensis* [8]. However, the morphological characters used to describe these species, such as colour and elytra, were not sufficient to accurately differentiate between them as distinguishing the striped form of *A. sexvittatus* from *A. oleae* has proved to be challenging. Therefore, additional morphological characters such as aedeagus and spermatheca shape, length and width of the elytra stripes, and colour and pronotal punctuation are helpful to identify *A. capensis*, *A. oleae* and *A. sexvittatus* (M. Biondi, personal data). *Argopistes sexvittatus* and *A. capensis* were originally described from the Western Cape province of South Africa, while *A. sexvittatus* was described from the Western and Eastern Cape, KwaZulu-Natal and the Free State provinces [7,8]. *Argopistes sexvittatus* currently has a wider distribution as it has been reported from various other localities in South Africa, the largest producer of olives and olive-derived products in southern Africa, and Namibia [9].

The larvae of both Asian and African *Argopistes* are leaf miners, and *A. sexvittatus* was reported as the most important pest of olive trees by J. Sneid Taylor in Graaff-Reinet (Eastern Cape) in 1945 [10]. The life histories of *A. capensis* and *A. oleae* have not been studied, but it is assumed that they would be similar to that of *A. sexvittatus*. According to Taylor (1945) and Myburgh (1952), *A. sexvittatus* females lay eggs singly or in clusters of up to ten eggs in cavities chewed into the epidermis, mostly on lower but also upper leaf surfaces [10,11]. Each elongate yellow egg is enclosed in a cell of hardened excrement. In spring, eggs hatch in about three weeks and in two weeks during summer. Larvae mine under the epidermis, mostly on the lower leaf surfaces, and prefer young foliage. They are often seen crawling on the leaf surface when moving to a fresh leaf. As the larvae grow, they are easily noticed as yellow bulges that give the leaves a blistered appearance. Larval development takes about three weeks in spring, probably less in summer. Mature larvae (approximately 7 mm long) drop to the ground and construct circular cells in which they pupate. Adult beetles emerging from the soil are soft and yellow in colour, but the exoskeleton soon hardens and darkens. Myburgh (1952) reported that overwintering adults in the Western Cape begin ovipositing from August to late October. It appears that there are three generations per year, with adults of the third brood, the overwintering generation, emerging at the end of March or the beginning of April. During cold weather, the overwintering adults become lethargic, while they feed sporadically on warmer days [11]. Olive flea beetles have not been reported in the Mediterranean and California, the main olive-producing regions in the world. *Lythraria salicariae* (Paykull, 1800) was found on cultivated olive orchards in Latvia and Turkey; however, it was not possible to conclude that olive trees were their host plant, as feeding and ovipositing were not observed [4,12].

This work is part of a larger effort to catalogue the entomofauna associated with African Wild Olive and European cultivated olive trees in South Africa. For that purpose, we aimed to identify olive flea beetle species in the Western Cape and assess their distribution in the province, and to determine their phylogenetic position in the tribe Alticini using new mitochondrial genomes.

## 2. Materials and Methods

### 2.1. Specimen Collection, Morphological Identification and DNA Extraction

**Figure 1:**
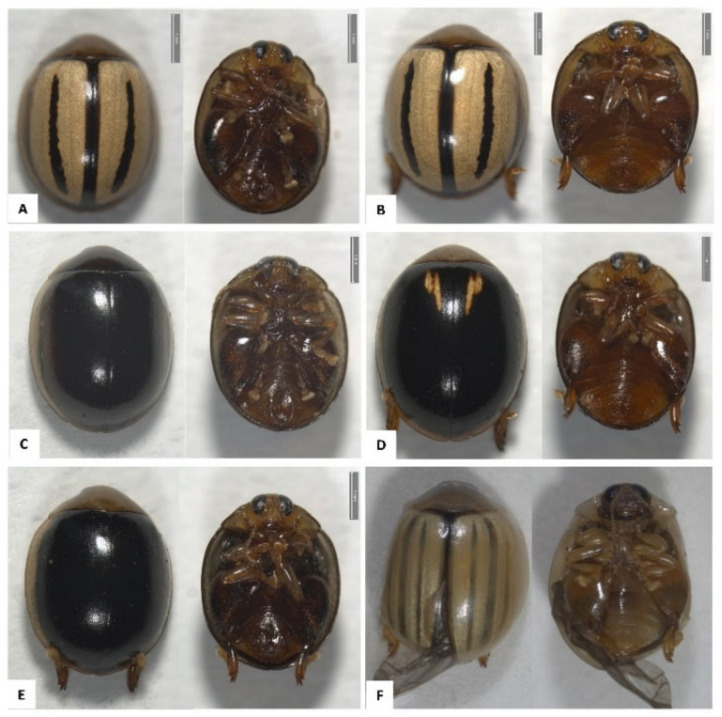
Representative adult specimens of olive flea beetles (Chrysomelidae: Alticini) species found in the Western Cape of South Africa. (**A**) *Argopistes sexvittatus*, black morphotype (male); (**B**) *A. sexvittatus*, black morphotype (female); (**C**) *A. sexvittatus*, black morphotype (male); (**D**) *A. sexvittatus*, striped morphotype (male); (**E**) *A. sexvittatus*, striped morphotype (female); (**F**) *Argopistes capensis* (female). Image credits: Elizabeth Grobbelaar.

**Figure 2:**
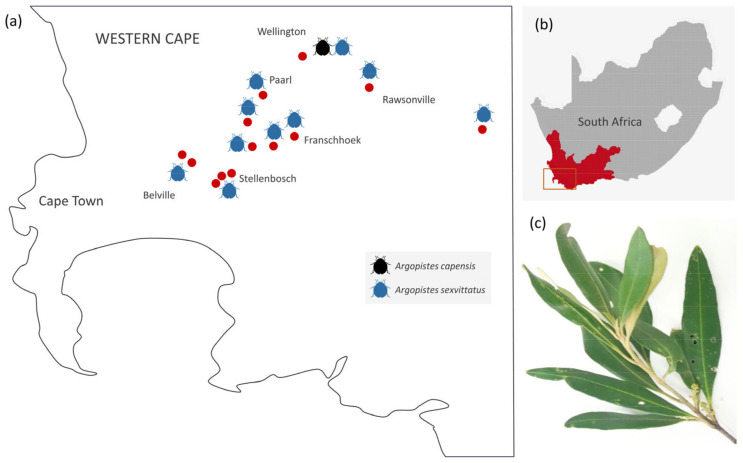
(**a**) Distribution of *A. capensis* and *A. sexvittatus* found in African Wild Olive and European cultivated olive trees in the Western Cape province of South Africa. Red dots indicate the approximate location of sampling sites; (**b**) Study area in the Western Cape; (**c**) Characteristic leaf damage caused by feeding of olive flea beetles.

Olive flea beetles (*Argopistes*) are characterized by their oval shape, 4 to 5 mm in length, and their heads are usually hidden below the thorax. Species are distinguishable by the colouration and the pattern of the elytra. The striped morphotype of *A. sexvittatus* has yellow elytra with generally wide, dark brown, median longitudinal, inner stripes with lateral margins (Figure 1A,B). *Argopistes sexvittatus* can also have entirely black/brown elytra and thorax (Figure 2C,E). The stripes on the yellow elytra of *A. capensis* are longitudinal, dark brown, faint, and irregular (Figure 1F). Two adult specimens of *A. sexvittatus* (one black morphotype and one stripped morphotype) were deposited in the insect collection of the Iziko Museums of South Africa in Cape Town with the codens SAM-COL-A095337 and SAM-COL-A095338 (SAMC; Curator Simon van Noort).

Adult olive flea beetles were collected from African Wild Olive and European cultivated olive trees at 14 locations in the Western Cape province of South Africa between November 2015 and March 2020 during studies focusing on other groups of olive-associated insects (Table S1). A specific survey of olive flea beetles on eight olive farms was conducted during the South African olive-growing season of 2020 (Figure 2). Only olive trees which exhibited typical symptoms of olive flea beetle infestation, such as holes on the leaves and desiccated leaf tips, were sampled. The number of olive trees surveyed at each farm varied from 10 to 50, and every second tree was sampled in any given block. Olive flea beetles (*n* = 291) were collected directly into plastic bags by hand, euthanized by freezing, and individually preserved in 100% ethanol at −4 °C. Morphological identification of ethanol-preserved specimens was performed by co-author M. Biondi, an expert on African flea beetles. Total DNA was extracted from a subset of the total specimens collected (*A. sexvittatus*, *n* = 31; *A. capensis*, *n* = 1) using a standard phenol-chloroform method [13] and stored at −4 °C until downstream analyses.

### 2.2. DNA Barcodes

The standard barcoding region of the mitochondrial *COI* gene was amplified and sequenced using the universal arthropod primers LCO1490 5′-GGTCAACAAATCATAAAGATATTGG-3′ and HCO 2198 5′-TAAACTTCAGGGTGACCAAAAAATCA-3′ [14]. PCR amplifications were performed in a total volume of 5 μL containing 1x KAPA2G Robust HotStart Ready Mix PCR kit (KAPPA Biosystems), 0.5 μM of each primer, 0.5 μL of MilliQ H_2_O and 1.0 μL of template DNA (~100 ng/μL). The thermal cycling program consisted of 95 °C for 3 min; 35 cycles at 94 °C for 15 s, 54 °C for 15 s, 72 °C for 1 min; and a final extension at 72 °C for 1 min. PCR products were sequenced using the reverse PCR primer with the BigDye Terminator v3.1 Cycle Sequencing Kit (Applied Biosystems, Waltham, MA, USA) at the Central Analytical Facilities of Stellenbosch University, South Africa.

Multiple sequence alignments were performed using the MAFFT algorithm [15] in Geneious Prime v2021.1 (www.geneious.com), accessed on 1 September 2022. Intra- and interspecific genetic divergences were estimated as pairwise distances (max p-distance, %) on MEGA X [16] under the Kimura 2-parameter model [17], accessed on 1 September 2022. A median-joining network of DNA barcode haplotypes of *A. sexvittatus* was constructed with Network 10.2 software under the default settings [18].

### 2.3. Mitogenomics

Two specimens of *A. sexvittatus* (striped and black morphotypes) and one specimen of *A. capensis* were sequenced using the Ion Torrent™ S5™ platform (ThermoFisher Scientific, Waltham, MA, USA) available at the Central Analytical Facilities of Stellenbosch University. Sequence libraries were prepared using the Ion Xpress™ Plus gDNA Fragment Library Kit (ThermoFisher) according to the protocol MAN0009847 REV J.0. Libraries were pooled and sequenced using the Ion 540™ Chef Kit (ThermoFisher). The high-throughput sequencing (HTS) reads for each specimen were separately mapped against the complete mitogenome of *Argopistes tsekooni* Chen (NC_045929.1) [19] and assembled with Geneious Prime. Open reading frames of protein-coding genes (PCGs) were identified with Geneious Prime using the invertebrate mitochondrial genetic code. Transfer RNA genes (tRNAs) and their secondary structures were predicted using the ARWEN software (http://130.235.244.92/ARWEN/) [20], accessed on 20 September 2022. The two ribosomal RNA genes (12S rRNA and 16S rRNA) and the putative control region (AT-rich region) were inferred and manually annotated by comparison with the mitogenomes of other Alticini. Start codons, and overlapping and intergenic spaces were counted manually. Nucleotide composition and compositional biases were calculated in Geneious Prime [AT-skew = (A − T)/(A + T) and GC-skew = (G − C)/(G + C)]. Relative synonymous codon usage was calculated in MEGA X. Ratios of nonsynonymous (Ka) to synonymous (Ks) substitutions (Ka/Ks) were calculated in DnaSP6 v6.12.03 (http://www.ub.edu/dnasp/), accessed on 20 September 2022 [21].

### 2.4. Phylogenetics

The phylogenetic position of *A. capensis* and the two morphotypes of *A. sexvittatus* was assessed in the context of 61 other mitogenomes for Alticini available on GenBank as of 1 August 2022, with *Aeolesthes oenochrous* (Cerambycinae), *Anoplophora glabripennis* (Lamiinae), *Crioceris duodecimpunctata* (Criocerinae) and *Phaedon tumidulus* (Chrysomelinae) as outgroups (Table S2). At the time of our analyses, approximately 110 mitogenomes were available for Alticini, but some genera were overrepresented (e.g., *Longitarsus*, 18 species; *Psylliodes*, 14 species; *Phyllotreta*, eight species; *Chaetocnema*, eight species). Therefore, we opted to use a maximum of two species from each genus to avoid potential skews in the phylogenetic estimates. Individual PCGs were extracted from each mitogenome and aligned using the translation algorithm in Geneious Prime. Stop codons were removed manually, and individual gene alignments were concatenated in a single alignment. Poorly aligned regions and gaps in the concatenated alignment were excluded using GBlocks v0.91b (https://ngphylogeny.fr/tools/tool/276/form) [22], accessed on 25 September 2022. A maximum likelihood (ML) tree was constructed based on the nucleotide sequence alignment using IQ-Tree 2.2.0 (http://www.iqtree.org/) [23] on the web server of CIBIV, Austria, accessed on 25 September 2022. Model selection was automatically determined [24] using 1000 replicates for both Ultra-Fast Bootstrap [25] and Sh-aLRT support [26]. The best fit model was GTR + F + I + G4, according to Bayesian Information Criterion. Bayesian analysis was performed under the site-heterogeneous mixture model CAT + GTR in PhyloBayes MPI v1.9 [27]. Constant sites were removed from the alignment, and the minimum number of cycles was set to 30,000, with the burn-in set to 1000. The “maxdiff’’ was set to 0.3, and the minimum effective size was set to 50. The final trees were drawn using iTOL v5 [28]. The DNA barcodes and complete mitogenomes of *A. capensis* and *A. sexvittatus* generated in this study were deposited on GenBank under the accession numbers OP858961 to OP858989, and OP868831 to OP868831, respectively.

## 3. Results and Discussion

Olive flea beetles are found feeding on *O. europaea* in southern Africa, especially in the Western and Eastern Cape provinces of South Africa, with *A. capensis*, *A. oleae* and *A. sexvittatus* associated with African Wild Olive and European cultivated olive trees. In this regard, it must be mentioned that a study performed in the Eastern Cape, where European-cultivated olive trees are not common, reported an additional three new species, *Argopistes epomistus*, *Argopistes lilliputiamus* and *Argopistes melanus*. However, these species were only described in a doctoral thesis; thus, according to article 9, point 9.12 of the International Code of Zoological Nomenclature [29], the three names cannot be considered valid. Furthermore, *A. epomistus* and *A. melanus* are the melanic chromatic forms of *A. sexvittatus* and, therefore, synonyms of this species (M. Biondi, personal data).

### 3.1. Distribution of Olive Flea Beetles in the Western Cape of South Africa

In the present study, *A. sexvittatus* was the dominant species as only two specimens of *A. capensis* were found at one site, and *A. oleae* was not found over a period of five years (2015–2020). Myburgh (1952) studied the bionomics and control of olive flea beetles in the Stellenbosch and Groot Drakenstein areas in the Western Cape over a period of 10 years and also reported that no *A. oleae* were collected [11]. The reasons for the apparent rarity of *A. capensis* and *A. oleae* in the areas sampled during this study are unknown. It is possible that the recent severe drought, which impacted hugely cultivated and wild olives, as well as widespread applications of broad-spectrum insecticides to control olive lace bugs (Hemiptera: Tingidae) and olive flea beetles, may have affected populations of *A. capensis* more severely than those of *A. sexvittatus*. However, neither Taylor (1945) [10] nor Myburgh (1952) recorded *A. capensis* in their surveys, although the latter mentions a small green beetle that does not match the description of *A. capensis* [11]. Thus, in practical terms, *A. sexvittatus* seems to be the only olive flea beetle currently affecting African Wild Olive and European cultivated olive trees in the Western Cape, as reported by Myburgh (1952) [11].

Formal questionnaires were not administered, but some olive farmers stated that insecticide spraying is sometimes necessary to control olive flea beetles, in which olive trees are sprayed twice a year. Severe infestations may warrant the use of insecticides up to four times per year to significantly reduce populations. Managing olive flea beetle infestations with insecticides is costly, and excessive spraying increases the risk of pests developing resistance to the treatment. The range of known natural enemies of olive flea beetles is currently limited to *Pseudophanomeris inopinatus* Belokobylskij (Hymenoptera: Braconidae), a parasitoid of *Argopistes* larvae in South Africa [30]. However, the rate of parasitism was low, and further surveys for finding natural enemies of olive flea beetles have not been performed.

### 3.2. DNA Barcodes Support Species Identification in A. capensis and A. sexvittatus

**Figure 3:**
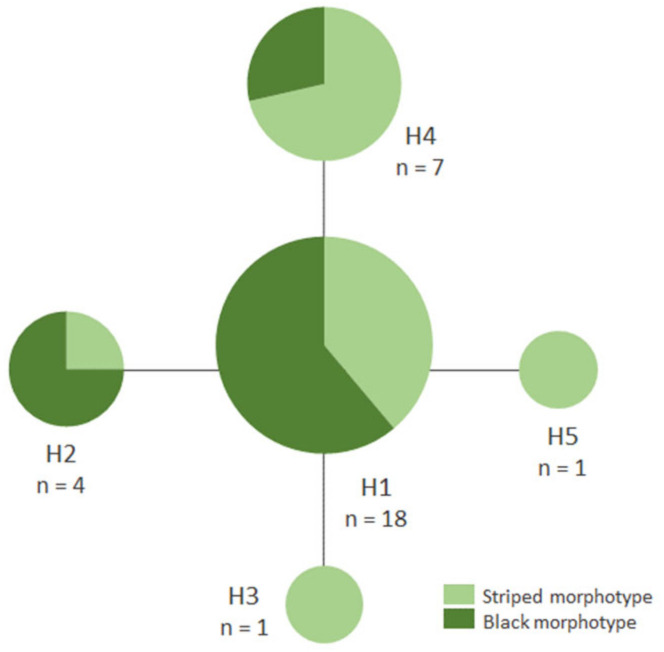
Median-joining network of DNA barcode haplotypes of *Argopistes sexvittatus* (Chrysomelidae: Alticini) (*n* = 31).

Genetic divergence based on DNA barcodes was consistent with morphological identification. The intraspecific genetic divergence of *A. sexvittatus* (*n* = 31; max p-distance = 0.31%) is compatible with the conspecificity of specimens, and the divergence between *A. sexvittatus* and *A. capensis* supports that they belong to different species (interspecific max p-distance = 10.43%); however, this result must be approached with caution as only one sequence was generated for *A. capensis* due to inadequate storage of the second specimen. For the same reason, it was not possible to gain insights into the intraspecific genetic divergence in this species. The four haplotypes of *A. sexvittatus* (striped and black) are very similar and separated by one mutation step (Figure 3), and are neither structured phylogeographically across the sampling areas nor between wild and cultivated hosts.

### 3.3. The Mitogenomes of Argopistes capensis and Argopistes sexvittatus

**Figure 4:**
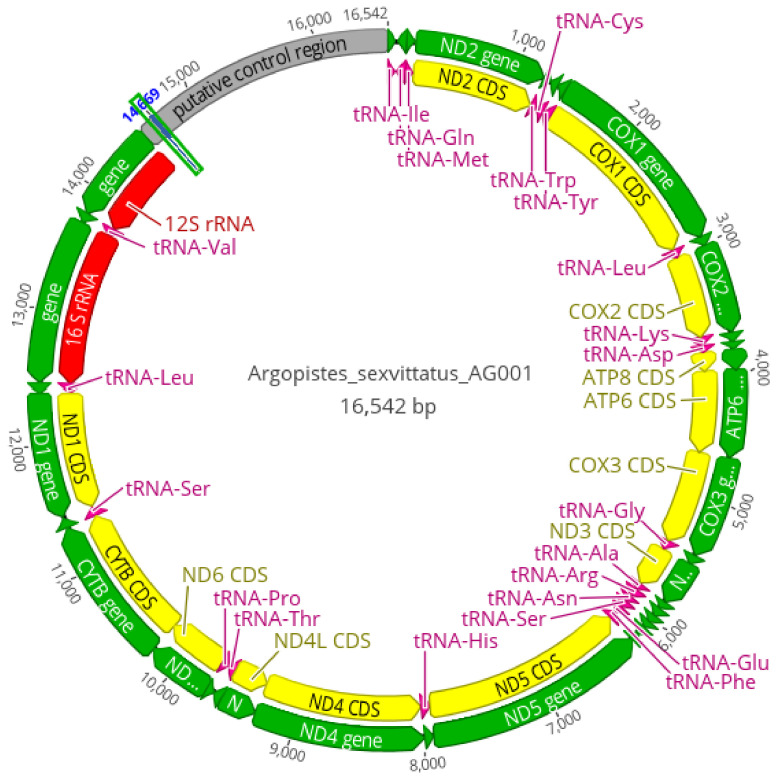
Circular map of the complete mitochondrial genome of *Argopistes sexvittatus* and *A. capensis,* here represented by *A. sexvittatus* AG001. Arrows indicate the direction of gene transcription.

The Ion Torrent sequencing runs generated 8 million reads with an average length of 174 bp for *A. capensis*, 13.7 million reads (162 bp) for *A. sexvittatus* (striped morphotype), and 15.9 million reads (160 bp) for *A. sexvittatus* (black morphotype). A total of 5116 reads from *A. capensis*, 113,345 reads from *A. sexvittatus* (striped), and 1.1 million reads from *A. sexvittatus* (black) were mapped to the reference sequence (*A. tsekooni*). The final sequence coverage of *A. capensis*, *A. sexvittatus* (striped) and *A. sexvittatus* (black) was 52×, 653× and 371×, respectively, exceeding the minimum usually required (15×) for mitogenomic studies [31]. The average mitogenome length was similar to other Alticini (16,009 bp), and the typical set of 37 mitochondrial genes found in metazoans [32] was identified, including 13 PCGs, 22 transfer tRNAs, two rRNAs, and the AT-rich region presumed to contain the initiation of transcription and replication [33]. Mitogenome architecture and gene size were identical in the three mitogenomes, represented by *A. sexvittatus* (striped morphotype) in Figure 4. Nine PCGs and 14 tRNAs are located on the majority (J) strand, and four PCGs, eight tRNAs and two rRNAs are encoded on the (N) minority strand (Table S3). Gene arrangement seems to be conserved in Alticini and is identical to the hypothetical ancestral organization for Arthropoda [32] in all Alticini species included in the mitogenomic analyses.

#### 3.3.1. Transfer RNAs, Ribosomal RNAs and AT-Rich Region

**Figure 5:**
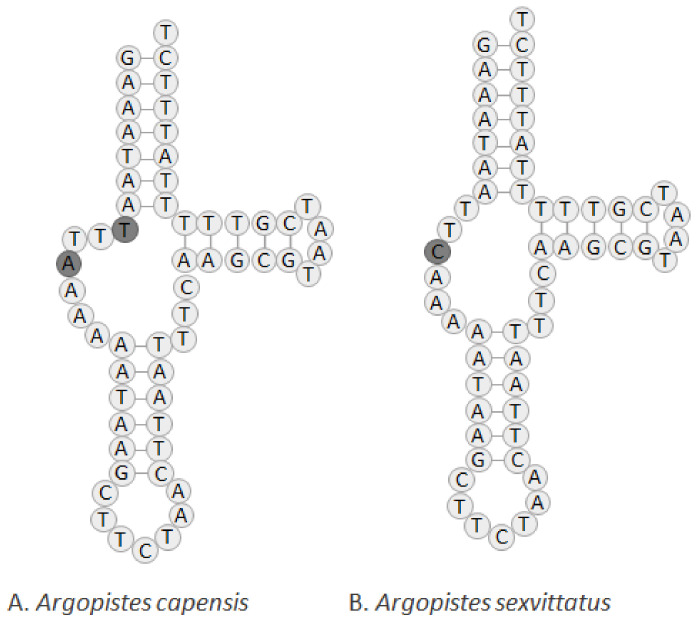
Predicted structure of tRNA^Ser1(TCT)^ in the complete mitochondrial genomes of (**A**) *A. capensis* and (**B**) *A. sexvittatus* (striped and black morphotypes).

The set of tRNA genes identified with ARWEN was manually compared to those found in other Alticini, and the most probable 22 tRNAs were annotated. Typical cloverleaf-like structures were predicted for all tRNAs except tRNA^Ser1(TCT)^, in which the dihydrouridine (DHU) arm is reduced and replaced by a simple loop (Figure S1), a common feature of metazoan mitogenomes [34]. *Argopistes capensis* and *A. sexvittatus* differ in the nucleotide sequence of tRNA^Ser1(TCT)^, including an additional nucleotide in *A. capensis* (Figure 5). This feature was also observed in other Alticini species, such as *Agasicles hygrophila* (Selman and Vogt 1971) [35]. The length of tRNAs ranges from 59 bp (tRNA^Ser1^) to 72 bp (tRNA^Lys^) in *A. capensis*, and 58 bp (tRNA^Ser1^) to 72 bp (tRNA^Lys^) in *A. sexvittatus*. The 16S rRNA gene is located between tRNA^Leu1^ and tRNA^Val^, and the 12S rRNA is located between tRNA^Val^ and the AT-rich region. The length of 16S rRNA is 1278 bp in *A. capensis* and *A. sexvittatus* (striped) and 1279 bp in *A. sexvittatus* (black), in line with other Alticini (average = 1220 bp). The 12S rRNA is 738 bp for *A. capensis* and *A. sexvittatus*, also in line with other Alticini (average = 756 bp). The large non-coding region (AT-rich region) is located between the 12S rRNA and the I-Q-M tRNA cluster and is similar in the two species (average = 2019 bp) and within the range found in other Alticini, which varies between 1309 bp in *Macrohaltica subplicata* (Leconte 1859) and 2020 bp in *A. tsekooni*.

#### 3.3.2. Start Codons and Stop Codons

**Figure 6:**
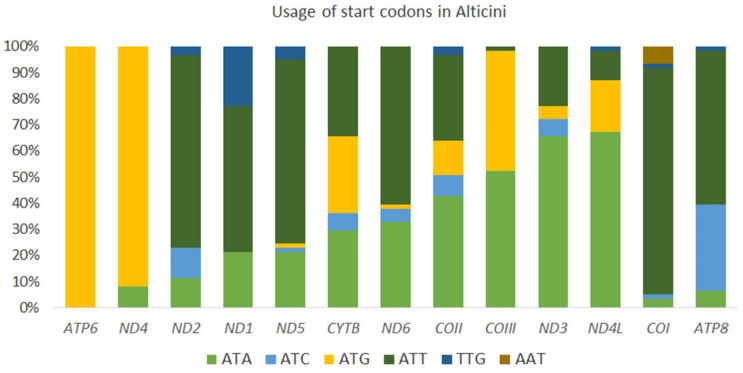
Usage of start codons found in the complete set of mitochondrial protein-coding genes in 64 species in the tribe Alticini (Coleoptera: Chrysomelidae).

Most PCGs in Alticini start with ATN, except *ND1* in 14 species, *COI* in four species, *ND5* in three species, *COII* and *ND2* in two species, and *ATP8* and *ND4L* in one species, all of which used alternative start codons GTG and AAT. The most frequently used start codon is ATG, and the least frequently used is AAT (Figure 6). All PCGs in *A. capensis* and *A. sexvittatus* use ATN start codons: ATG (*ATP6*, *COII*, *COIII*, *CYTB*, *ND4* and *ND4L*, ATT in *ND2*, *ND3*, *ND4*, *ND5* and *ND6*) and ATC (*ATP8*), except for *COI* (AAT) and *ND1* (TTG). TTG is a start codon in other mitochondrial PCGs in Coleoptera, including *Altica cirsicola* Ohno, *Altica fragariae* Nakane and *Altica viridicyanea* (Baly) [36], and *A. tsekooni* [19]. Most PCGs of *A. capensis* and *A. sexvittatus* terminate with TAA, except *ND1*, which terminates with TAG. Incomplete stop codons (TA and T) are present in *A. capensis* and *A. sexvittatus* (*COI*, *COII*, *COIII*, *CYTB*, *ND2*, *ND3*, *ND4* and *ND5*) and are generally presumed to be completed by posttranscriptional polyadenylation [37,38].

#### 3.3.3. Intergenic Regions and Spacers

Both olive flea beetle species have highly compact mitogenomes, with short intergenic spaces present at four locations in *A. capensis* (total of 22 bp) and at five locations in *A. sexvittatus* (total of 33 bp). Intergenic spacers range between 1 and 17 bp, with the longest located between *ND1* and tRNA^Ser2^ in both species, in line with *A. tsekooni* (17 bp). The total number of intergenic nucleotides in other Alticini ranges from 1 to 61 bp. *Argopistes capensis* has a total of 19 gene overlaps, and *A. sexvittatus* has 18, mostly involving tRNAs. The longest overlap in both species (10 bp) is between tRNA^Cys^ and tRNA^Trp^, which are encoded in opposite directions, and *ND4L*-*ND4* and *ATP8*-*ATP6* overlap by 7 bp, as in *A. tsekooni*, and in line with other Alticini (average 11 bp).

#### 3.3.4. Nucleotide Composition and Codon Usage

*Argopistes capensis* and *A. sexvittatus* have the high A + T content typically found in insect mitogenomes (Table S4). The A + T content of the AT-rich regions of the mitogenomes (*A. capensis*, 90.5%; *A. sexvittatus* striped, 85.7%; *A. sexvittatus* black, 86.9%) is higher than that of their complete sequences (average = 80%). The A + T content of the combined rRNAs in *A. capensis* (82.9%) and *A. sexvittatus* (82.8%) is similar, as well as total A + T content in PCGs (*A. capensis* = 78.2%; *A. sexvittatus* = 78.5%). ATP8 has the highest A + T content in both species (*A. capensis* = 90.3%; *A. sexvittatus* striped = 87.0%; *A. sexvittatus* black = 87.4%), and *COI* has the lowest (A + T = 71.6%). The three mitogenomes have negative AT-skew and negative GC-skew in most regions (PCGs, tRNAs, rRNAs and AT-rich region) except *ATP8*, *ND1*, *ND4*, *ND4L* and *ND5*, as well as *COII* in *A. sexvittatus*. In both species, four of 13 PCGs on the N-strand have higher AT-skew values than PCGs on the J-strand. The nucleotide bias towards A and T is reflected in codon usage, with AT-rich codons (UUU, UUA, AUU, AUA, UAU, AAU and AAA) representing 42.3% of all codons in *A. capensis*, 51.5% in *A. sexvittatus* (striped) and 52.1% in *A. sexvittatus* (black). Relative synonymous codon usage (RSCU) is calculated as the relative frequency of a codon within a mitogenome. An RSCU value > 1.0 indicates an over-represented codon, whereas an RSCU value < 1.0 indicates an under-represented codon [39]. RSCU is higher than 1.0 among all synonymous codons, indicating that AT-rich codons are favoured among synonymous codons (Table S5). Nucleotide composition, AT-skew and codon usage in *Argopistes* species (including the mitogenomes of *A. tsekooni* and one *Argopistes* sp. available on GenBank) is also unremarkable and similar to other Alticini.

#### 3.3.5. Synonymous and Nonsynonymous Nucleotide Substitution Rates

**Figure 7:**
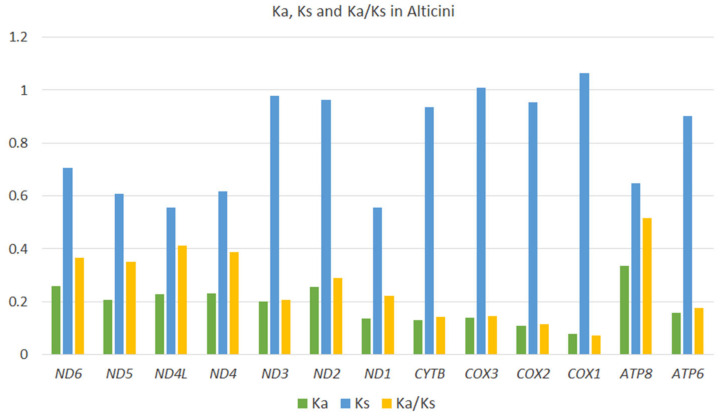
Evolutionary rates (Ka/Ks) of 13 mitochondrial protein-coding genes of 64 species of Alticini (Coleoptera: Chrysomelidae).

The Ka/Ks ratio of non-synonymous (Ka) to synonymous (Ks) nucleotide substitutions is used as an indicator of selective pressure on protein-coding sequences among different sequences [40]. Ka/Ks ratio > 1 indicates positive selection, which is assumed to have occurred during the evolution of the sequence. Average Ka/Ks was calculated for individual PCGs across the 64 Alticini species included in this study. All genes have Ka/Ks < 1, indicating evolution under purifying selection. *COI* has the lowest Ka/Ks (0.07), and *ATP8* has the highest (Ka/Ks = 0.51) (Figure 7).

### 3.4. Mitogenomic Variation of Argopistes sexvittatus Morphotypes

The complete mitogenomes of the two morphotypes of *A. sexvittatus* (striped and black) differ in 3.3% of the total sequence. In the 13 PCGs, the two morphotypes differ by 0.17% of the sequence with 19 single nucleotide polymorphisms (SNPs), all of which are synonymous substitutions. *ND1*, *ND3*, *ND4L* and *ND6* are completely conserved between the two morphotypes, and *CYTB* and *ND4* are the most polymorphic genes with five and four SNPs, respectively. Therefore, the two morphotypes can be considered genetically identical at the level of mitochondrial DNA.

### 3.5. Phylogenetic Position of A. capensis and A. sexvittatus within Alticini

**Figure 8:**
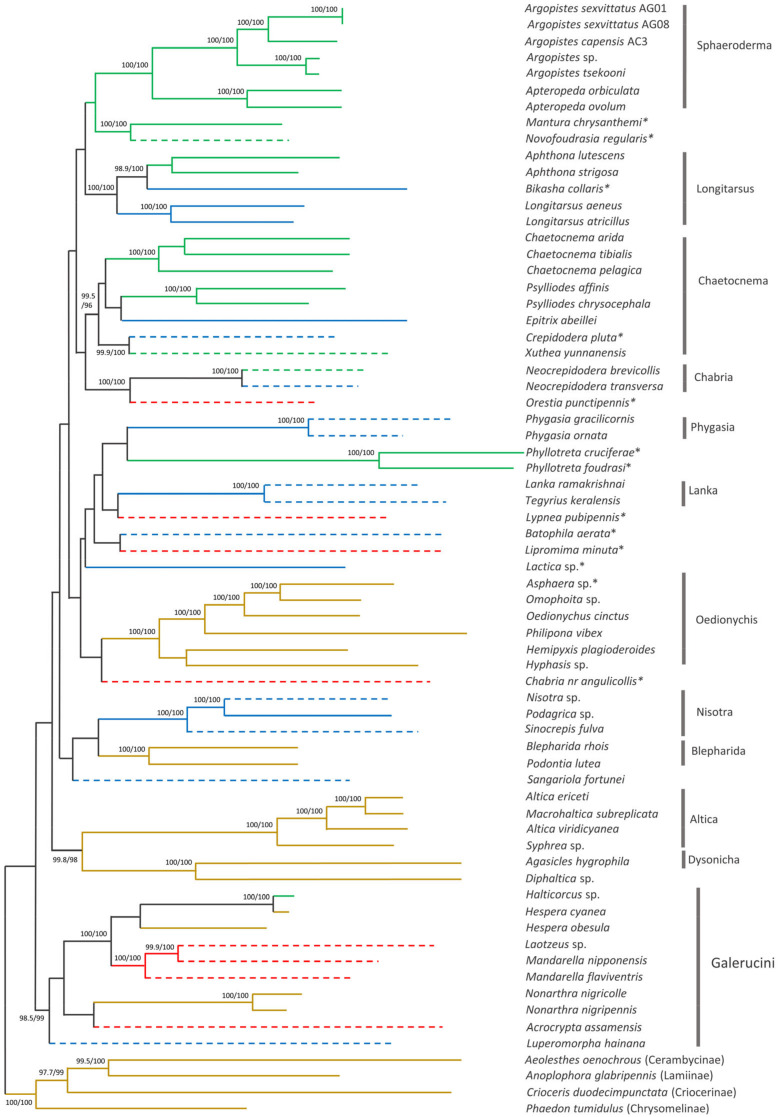
Maximum likelihood phylogenetic tree of 64 Alticini species based on 13 mitochondrial protein-coding genes. Nodal support is given as SH-aLRT (%)/ultrafast bootstrap (%); only values >95% are shown. Branch colours represent larval feeding habits: leaf miner—green; external leaf feeder—brown; root feeder—blue; unknown—red. Dashed branches represent possible/unconfirmed/unclear larval feeding habits. * Genus group unclear.

**Figure 9:**
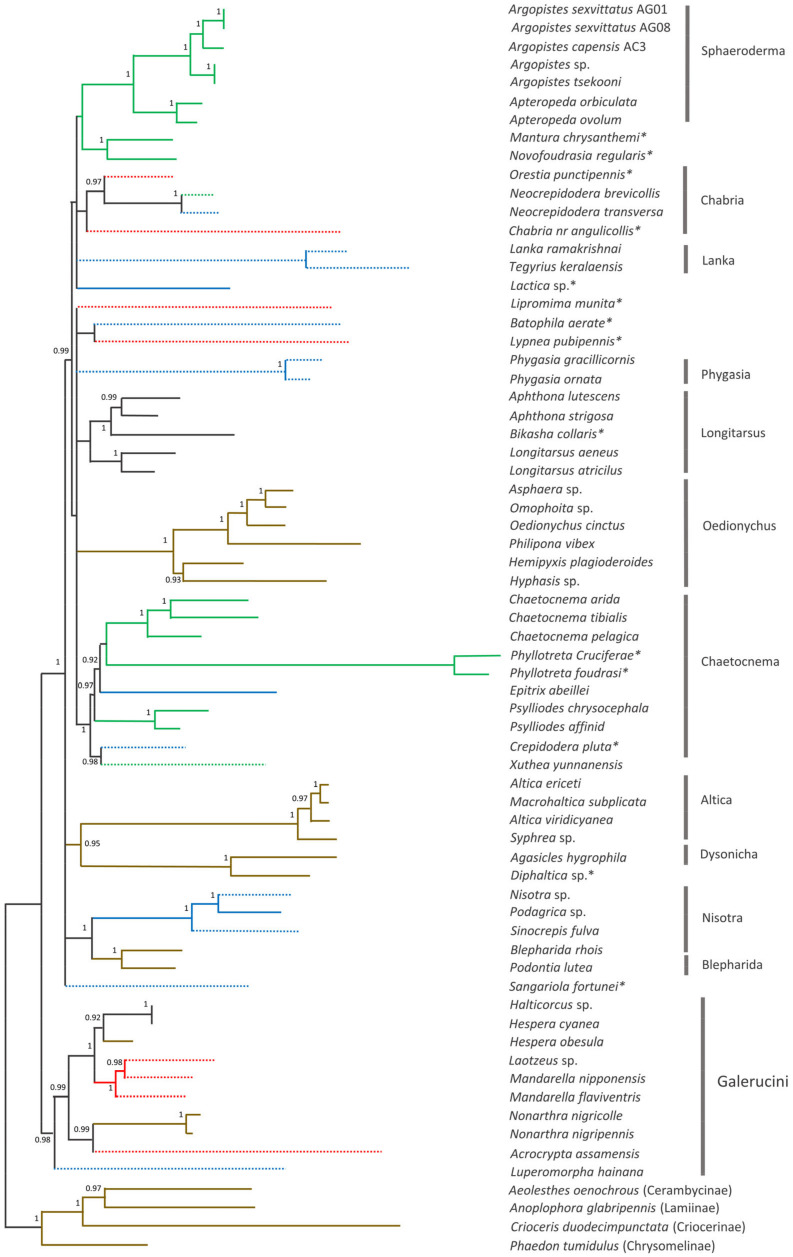
PhyloBayes tree of 64 Alticini species based on 13 mitochondrial protein-coding genes. Nodal support is given as Bayesian Posterior Probability; only values > 0.90 are shown. Branch colours represent larval feeding habits: leaf miner—green; external leaf feeder—brown; root feeder—blue; unknown—red. Dashed branches represent possible/unconfirmed/unclear larval feeding habits. * Genus group unclear.

The phylogenetic position of *A. capensis* and *A. sexvittatus* in Alticini was recovered using 64 other mitogenomes available for the tribe on GenBank. The ML tree has high statistical support (SH-aLRT ≥80% and UFBoot ≥ 95%) [26] for a large proportion of the more recent nodes, but the deeper nodes generally have low support (Figure 8). The PhyloBayes tree follows the same pattern with high nodal support (BPP = 1) for most of the recent nodes, but some of the deeper nodes are unresolved (Figure 9). The monophyletic groups recovered by Nie et al. (2018) [41] are broadly present in our phylogeny, albeit in different order of the deeper nodes. This is likely due to the different genetic datasets utilized in the analyses, as our work relies exclusively on the 13 mitochondrial genes, and the previous study combined mitochondrial and nuclear markers. Moreover, we included 22 additional Alticini species not used in Nie et al. (2018) [41]. Nonetheless, the “problematic genera” (*Mandarella*, *Laotzeus*, *Hespera, Halticorcus*, *Nonarthra* and *Acrocrypta* that cannot be classified in Alticini or Galerucini based on the presence of the jumping apparatus appear in basal to all other Alticini, in agreement with Nie et al. (2018) [41] where these genera grouped in Galerucini.

All “non-problematic” genus groups represented by more than one species appear monophyletic with high statistical support (*Sphaeroderma*, *Phygasia*, *Lanka*, *Nisotra*, *Blepharida*) in both our trees. Some inconsistencies were evident: *Bikasha collaris* (*Aphthona* group) falls within the *Longitarsus* group; *Lipromima minuta* falls outside its attributed *Chaetocnema* group; *Diphaltica* sp. falls outside its attributed *Oedionychis* group. The *Chabria* group recovered in Nie et al. (2018) [41] is not monophyletic in our ML tree, as *Chabria* nr. *angulicollis* and *Neocrepidodera* appear in different branches; this inconsistency was also highlighted by Damaška et al. (2021) [42]. However, *Chabria* nr. *angulicollis* groups with *Neocrepidodera* in the PhyloBayes tree, but the *Chabria* group is not monophyletic due to *Orestia punctipennis* (genus group unknown) being placed within it. *Altica* forms a cluster with *Macrohaltica* as seen previously [41], and *Batophila aerata* (genus group unclear) is placed in the *Altica* group in Nie et al. (2018) [41] but not in our trees, and *Crepidodera pluta* (genus group unclear) falls within the *Chaetocnema* group. All *Argopistes* species fall within the monophyletic *Sphaeroderma* group with *Apteropeda*, here proposed as a member of this genus group for the first time.

Larval feeding habits (root feeder, leaf miner and external leaf feeder) are unclear, uncertain, or unconfirmed for a large proportion of the species included in the phylogeny estimates (39%; 25/64). Among the genera groups with confirmed larval feeding habit, only *Sphaeroderma* (leaf miner) and *Oedionychis*, *Altica* and *Blepharida* (external leaf feeders) show an association between phylogenetic cluster and larval feeding habit. Association between genera groups and host plant is not evident and different species in the same genus are often associated with different host plants in the same botanical family (Table S2). However, it is equally true that different species often share the same cultivated plants due to greater trophic availability. In our survey, we collected a slighter higher number of *A. sexvittatus* on European cultivated olive trees (*n* = 141) compared to African Wild Olive (*n* = 97), but we only found *A. capensis* once, so it is not possible to infer whether different species prefer different *O. europaea* hosts.

The taxonomic coverage of Alticini in our phylogenetic reconstruction is less than ideal due to the unavailability of mitogenomes for taxa closely related to *Argopistes*, especially *Dibolia* and *Jacobyana* [43]. Therefore, the phylogenetic position of *Argopistes* within Alticini could not be determined with high confidence. However, it is interesting to note how this genus (represented by *A. sexvittatus*, *A. capensis*, *A. tsekooni* and *Argopistes* sp.) forms a monophyletic clade sister to the Palearctic genus *Apteropeda*, which was not included in Nie et al. [41].

We have faced similar difficulties in determining the phylogenetic position of other African insects associated with *O. europaea* due to the paucity of mitogenomic data for closely related taxa. For example, the olive seed weevil *Anchonocranus oleae* (Coleoptera: Curculionidae) remains *incertae sedis* due to a lack of adequate taxonomic context [44]. Similarly, our study of the African olive lace bugs *Neoplerochila paliatseasi*, *Plerochila australis* and *Cysteochila lineata* (Hemiptera: Tingidae: Tingini) also had to rely on the presently limited taxonomic coverage of Tingini [45,46]. Interestingly, *C. lineata* (associated with *Olea*) clustered with the other olive-feeding lace bugs rather than with its congener *Cysteochila chiniana* (not associated with *Olea*), suggesting that the olive-feeding habit has a common evolutionary origin, at least at the level of the mitogenome, as seen in *Argopistes*. 

Compared to other agricultural crops, *O. europaea* is affected by a relatively small number of insect pests, and a large proportion of these are native to Africa, where they have co-evolved with specialized natural enemies [47,48]. Presently, native African insects associated with olives are practically absent in olive-growing regions worldwide, but an intensification of climate alterations and global trade routes may enable invasions. This was likely the case of the African citrus triozid, *Trioza erytreae* (Hemiptera: Triozidae), one of the most damaging pests of citrus pests in Africa for its ability to transmit the bacterial agent for African Citrus Greening disease, which is currently causing great concern due to its presence in northern Spain and Portugal [49]. Therefore, documenting the African entomofauna associated with *O. europaea* and other agricultural crops is of utmost importance.

## 4. Conclusions

Olive flea beetles can inflict serious damage on hosts and cause economic losses in commercial olive orchards. *Argopistes capensis*, *A. sexvittatus* and *A. oleae* have previously been found on African Wild Olive and European cultivated olive trees in the Western Cape, but systematic surveys had not been performed in over five decades. We found that *A. sexvittatus* is the dominant species in the region, while *A. capensis* is exceedingly rare, and *A. oleae* was not found over a period of five years. Furthermore, we generated mitogenomic and DNA barcoding data for *A. sexvittatus* and *A. oleae* with utility for documenting the diversity of olive-associated entomofauna in southern Africa. The phylogenetic position of *Argopistes* could not be estimated with high confidence due to a lack of adequate taxonomic context; however, the new and publicly available sequences for the genus formed a monophyletic cluster, in agreement with its specialization in Oleaceae hosts. The novel genetic and species distribution data are part of a larger ongoing effort to characterize the entomofauna associated with olives in southern Africa and will be useful for the identification and monitoring of these and other *Argopistes* species.

## Data Availability

The DNA sequences generated in this study were deposited on GenBank (DNA barcodes—accession numbers OP858961 to OP858989; mitogenomes—accession numbers OP868831 to OP868831.

## Supplementary Materials

The following supporting information can be downloaded at: https://www.mdpi.com/article/10.3390/genes13122195/s1, Table S1: Sample list; Table S2: Mitogenomes in phylogeny; Table S3: Mitogenome features; Table S4: Nucleotide composition; Table S5: Codon usage. Figure S1. Predicted secondary structure of tRNAs.
