# Supplementary material for: Argopistes sexvittatus and Argopistes capensis (Chrysomelidae: Alticini): Mitogenomics and Phylogeny of Two Flea Beetles Affecting Olive Trees
Source: Genes (Basel). 2022 Nov 23;13(12):2195. doi: 10.3390/genes13122195 (PMC9777630; doi:10.3390/genes13122195)
Supplement: Supplementary file 1 [file genes-13-02195-s001.zip › Figure S1 tRNAs.pdf]

Figure S1. Predicted secondary structure of 22 tRNAs in the complete mitogenome of *Argopistes sexvittatus.* All tRNAs are identical between *A. sexvittatus* and *Argopistes capensis* except tRNA$^{Ser(TCT)}$ (shown in Figure 5).

1. mtRNA-Ile(gat)

```
               a
             t-a
             a-t
             t-a
             a-t
             a-t
             g-c
             g+t    t
            t   caca
      ag   g    !!!! t
     t  tcc     gtgt
     t  !!!    t    t
      a agg     a
       a   a     c
            t-aat
            a-t
            a-t
            t-a
            t-a
           t   g
           t   a
            gat
```

2. mtRNA-Gln(ttg)

```
               a
             t-a
             t-a
             a-t
             t-a
             a-t
             t-a
             c-g
             t-a     tt
            t   ttatc  a
      a    a    !!!!!  a
     t tatg     aatag  t
     g +!!!     a    tt
     a gtac     g
      t    t    g
            t-aa
            t-a
            a-t
            a-t
            t-a
           t   t
           t   a
            ttg
```

3. mtRNA-Met(cat)

```
               a
             a-t
             a-t
             a-t
             a-t
             a-t
             g-c
             a-t     ta
            t   tttcc  a
      a    a    !!!!!  t
     a tcga     aaagg  t
     a !!!!     t    tt
     t agct     a
      a    a    t
            t ct
            t-a
            g-c
            g-c
            g-c
           t   c
           t   a
            cat
```

4. mtRNA-Trp(tca)

```
               a
             t-a
             a-t
             a-t
             g-c
             g-c
             a.g
             t-a
             t-a    t
            t   tctt
      a    a    !!!! t
     a ttga     agaa t
     a !!!!     t   a
     a aact     a
      a    t    a
            t-ac
            t-a
            a-t
            a-t
            c-g
           c   a
           t   a
            tca
```

5. mtRNA-Cys(gca)

```
               a
             a-t
             a-t
             g-c
             t+g
             c-g
             t-a
```

```
          t-a
          a-t    t
         t   tttc a
   a    a    !!!! a
  a actt     aaag t
  a !!!:     a   t
    tgat     g
   a    a    g
         t-at
         t-a
         a-t
         a-t
         a-t
        t   a
        t   a
         gca
```

6. mtRNA-Tyr(gta)

```
           a
          g-c
          a-t
          t-a
          a-t
          a-t
          a-t
          a-t    g
         t   ctta
   ag   g    !!!! a
  a  tcg     gaat t
 g   +!!     t   g
 t   ggc     a
  tta   a    t
         a-tt
         t-a
         a-t
         a-t
         a-t
        t   a
        t   a
         gta
```

7. mtRNA-Leu(taa)

```
            a
          t-a
          t-a
          c-g
          t-a
          a-t
          a-t
          t.t
          a-t    a
         t   tttc a
   ag   g    !!!!  a
  t  acg     aaag  t
 t   !!!     t   ct
 a   tgc     a
```

```
     aag   a    t
            t-aa
            t-a
            g-c
            g-c
            a c
           c   c
           t   a
            taa
```

8. mtRNA-Lys(ttt)

```
              a
             t-a
             c-g
             a-t
             t-a
             t-a
             a-t
             g-c
             a-t     agc
            t   ttata   a
      ag   g    !!!!!   a
     a  ccg     aatat  t
     a   !!    t     at
     g  agc     a
      ta   a     a
            a-ttt
            t-a
            g-c
            g+t
            a a
           a   a
           t   a
            ttt
```

9. mtRNA-Asp(gtc)

```
              a
             a-t
             a-t
             a-t
             a-t
             a-t
             t-a
             t-a     a
            t   taaaa
      a    a    !!!!! t
     a attg     atttt
     t !!!!     t    t
     t taac     t
      a    a    a
            t-aa
```

```
           t-a
           a-t
           g-c
           c-g
          t   a
          t   a
           gtc
```

10. mtRNA-Gly(tcc)

```
               a
            a-t
            t-a
            t-a
            t-a
            a-t
            t-a
            a-t    t
           t   gatt
    aa    a    !!!! a
   a  tata     ctaa t
   a  !!!!     t   c
    a atat     a
     a    a    a
           t-aa
           t-a
           t-a
           g-c
           a-t
          c   a
          t   a
           tcc
```

11. tRNA-Ala(tgc)

```
               a
            t-a
            a-t
            g+t
            g-c
            a-t
            t-a
            a-t
            a-t     t
           t   taact
     a    a    !!!!! t
    t attg     attga
    a !!!!     t    a
      taac     a
     a    a    a
           t-aa
           t-a
           t-a
           a-t
           a-t
          t   t
```

```
           t   a
            tgc
```

12. mtRNA-Arg(tcg)

```
                t
              a-t
              a-t
              a-t
              t-a
              a-t
              a-t
              g-c    t
             a   ctat
      a    a    !!!! t
     a aacg     gata a
     a !!!!    t    a
     t ttgc     t
      a    a     t
            t-aaa
            t-a
            t-a
            a-t
            g-c
           t   c
           t   a
            tcg
```

13. mtRNA-Asn(gtt)

```
                g
              t-a
              t-a
              a-t
              a-t
              t-a
              t-a
              g-c    a
             a   ctct
      aa   a    !!!! a
     a  cca     gaga
    c   !!!    t    t
    c   ggt     a
     cga   a     a
            t-att
            a-t
            t-a
            c-g
            a-t
           c   a
           t   a
            gtt
```

14. mtRNA-Ser(tct)

```
               t
             g-c
             a-t
             a-t
             a-t
             t-a
             a-t
             a-t     t
            t   tttgc
           t    !!+!! a
          c     aagcg a
          a     c    t
          a     t
           a    t
            a-tt
            a a
            t-a
            a-t
            a-t
            g-c
           c   a
           t   a
            tct
```

15. mtRNA-Glu(ttc)

```
                c
               t
             t-a
             a-t
             t-a
             t-a
             t-a
             a-t
             t-a
             a-t   t
            t   ttt t
      a    a    !!! a
     a tttg     aaa a
     a !!!!     t  t
     t aaac     a
      a    a    a
            t-aa
            t-a
            a-t
            c-g
            a-t
           t   a
           t   a
            ttc
```

16. mtRNA-Phe(gaa)

```
               t
             t-a
             a-t
             t-a
             c-g
             t-a
             a-t
             a-t
             a-t    a
            t   tcaa
      a    a    !!!! t
     t ttcg     agtt
     a +!!!     a   t
     a gagc     g
      a    a    g
            t-aa
            g+t
            a-t
            t-a
            a-t
           t   a
           t   g
            gaa
```

17. mtRNA-His(gtg)

```
               t
             a-t
             t-a
             c-g
             t-a
             a-t
             a-t
             a-t   t
            t   tat
      a    a    !!! t
     a tttg     ata t
     t !!!+     t  a
     a aaat     t
      a    a    g
            t-aa
            t-a
            g-c
            a-t
            t-a
           t   t
           t   a
            gtg
```

18. mtRNA-Thr(tgt)

```
                c
               t
             a-t
```

```
             g-c
             t-a
             t-a
             t-a
             t-a
             a-t
             a-t    t
            t   ttca
      a    a    !!!! a
     a ttta     aagt t
     a !!!!     t   a
     t aaat     a
      a    a    a
            t-aa
            t-a
            g-c
            g-c
            t-a
           t   a
           t   a
            tgt
```

19. mtRNA-Pro(tgg)

```
               a
             t-a
             c-g
             a-t
             g-c
             a-t
             t-a
             a-t
             t-a    t
            t   tctt
      a    a    +!!! a
     a tttg     ggaa a
     t !!!+     t   a
     t aaat     a
      a    a    g
            t-at
            t-a
            a-t
            a-t
            t-a
           t   g
           t   a
            tgg
```

20. mtRNA-Ser(tga)

```
               t
             a-t
             g-c
             t-a
```

```
             t-a
             t-a
             a-t
             t-a     t
            g   tcttt a
      g    a    !!!!! a
     t ttca     agaaa t
     t !!!!     a    t
     a aagt     a
      t    a    t
            t-aa
            a-t
            t-a
            a-t
            t-a
           t   a
           t   a
            tga
```

21. mtRNA-Leu(tag)

```
                c
               a
             a-t
             t-a
             t-a
             a-t
             t-a
             t-a
             t-a     t
            t   catta
      ag   g    !!!!! t
     a  acg     gtaat
     a  !!!     t    t
     a  tgc     a
      ag   a    t
            a-ta
            t.t
            a-t
            g-c
            a-t
           t   a
           t   a
            tag
```

22. mtRNA-Val(tac)

```
               t
             t-a
             c-g
             a-t
             a-t
             a-t
             t-a
             t-a
             a-t     t
```

```
       a   ttaac a
 a    a    !!!!!  a
  ttcg     aattg  a
t !!!!     t    tt
  aagc     t
 t    a    g
       t-aa
       t-a
       t-a
       t-a
       a-t
      g   t
      t   a
       tac
```
