# Supplementary material for: Argopistes sexvittatus and Argopistes capensis (Chrysomelidae: Alticini): Mitogenomics and Phylogeny of Two Flea Beetles Affecting Olive Trees
Source: Genes (Basel). 2022 Nov 23;13(12):2195. doi: 10.3390/genes13122195 (PMC9777630; doi:10.3390/genes13122195)
Supplement: Supplementary file 1 [file genes-13-02195-s001.zip › Table S1 Sample list.pdf]

Table S1. List of adult olive flea beetles of *Argopistes capensis* and *Argopistes sexvittatus* (Coleoptera: Chrysomelidae) collected in the Western Cape province of South Africa and used in this study for imaging, DNA barcoding and sequencing of complete mitochondrial genomes. Cultivated host: *Olea europaea* subsp. *europaea*; Wild host*: Olea europaea* subsp. *cuspidata.*

**Species**	**Specimen**	**Collection date**	**Host**	**Type of site/tree**	**Region**	**Latitude**	**Longitude**	**Use in this study**	**Genbank accession**
Argopistes capensis	AC02	10-Nov-15	Wild	Olive farm	Wellington	33°34'21.0"S	19°'03'39.0"E	Imaging	n.a.
Argopistes capensis	AC03	10-Nov-15	Cultivated	Olive farm	Wellington	33°34'21.0"S	19°'03'39.0"E	Mitogenome/Imaging	**Upon acceptance**
Argopistes sexvittatus	AB01	30-Dec-15	Cultivated	Ornamental tree	Stellenbosch	33°56'13.2"S	18°51'13.2"E	DNA barcode	**Upon acceptance**
Argopistes sexvittatus	AB02	13-Jan-16	Wild	Ornamental tree	Stellenbosch	33°55'34.0"S	18°51'53.0"E	Imaging	n.a.
Argopistes sexvittatus	AB03	13-Jan-16	Wild	Ornamental tree	Stellenbosch	33°55'34.0"S	18°51'53.0"E	DNA barcode	**Upon acceptance**
Argopistes sexvittatus	AB04	13-Jan-16	Wild	Ornamental tree	Stellenbosch	33°55'34.0"S	18°51'53.0"E	Imaging	n.a.
Argopistes sexvittatus	AB05	08-Nov-15	Wild	Olive farm	Wellington	33°34'21.0"S	19°'03'39.0"E	DNA barcode	**Upon acceptance**
Argopistes sexvittatus	AB06	30-Dec-15	Cultivated	Olive farm	Wellington	33°34'21.0"S	19°'03'39.0"E	DNA barcode	**Upon acceptance**
Argopistes sexvittatus	AE01	13-Jan-16	Wild	Ornamental tree	Stellenbosch	33°55'34.0"S	18°51'53.0"E	Imaging	n.a.
Argopistes sexvittatus	AE02	30-Dec-15	Cultivated	Ornamental tree	Stellenbosch	33°56'13.2"S	18°51'13.2"E	DNA barcode	**Upon acceptance**
Argopistes sexvittatus	AE03	13-Jan-16	Wild	Ornamental tree	Stellenbosch	33°55'34.0"S	18°51'53.0"E	Imaging	n.a.
Argopistes sexvittatus	AE05	10-Nov-15	Cultivated	Olive farm	Wellington	33°34'21.0"S	19°'03'39.0"E	DNA barcode	**Upon acceptance**
Argopistes sexvittatus	AE06	10-Nov-15	Cultivated	Olive farm	Wellington	33°34'21.0"S	19°'03'39.0"E	DNA barcode	**Upon acceptance**
Argopistes sexvittatus	AE09	16-Jan-16	Cultivated	Ornamental tree	Stellenbosch	33°56'13.2"S	18°51'13.2"E	DNA barcode	**Upon acceptance**
Argopistes sexvittatus	AG01	06-Feb-20	Cultivated	Ornamental tree	Stellenbosch	33°56'13.2"S	18°51'13.2"E	Mitogenome	**Upon acceptance**
Argopistes sexvittatus	AG08	06-Feb-20	Cultivated	Ornamental tree	Stellenbosch	33°56'13.2"S	18°51'13.2"E	Mitogenome	**Upon acceptance**
Argopistes sexvittatus	AG09	10-Feb-20	Cultivated	Ornamental tree	Stellenbosch	33°55'27.0"S	18°52'25.0"E	DNA barcode	**Upon acceptance**
Argopistes sexvittatus	AM01	13-Jan-16	Wild	Ornamental tree	Stellenbosch	33°55'34.0"S	18°51'53.0"E	DNA barcode	**Upon acceptance**
Argopistes sexvittatus	AM05	10-Nov-15	Wild	Ornamental tree	Stellenbosch	33°55'34.0"S	18°51'53.0"E	DNA barcode	**Upon acceptance**
Argopistes sexvittatus	AM35	30-Dec-15	Cultivated	Ornamental tree	Stellenbosch	33°56'13.2"S	18°51'13.2"E	Imaging	n.a.
Argopistes sexvittatus	AM42	17-Jan-16	Cultivated	Olive farm	Wellington	33°34'21.0"S	19°'03'39.0"E	DNA barcode	**Upon acceptance**
Argopistes sexvittatus	AM45	10-Nov-15	Cultivated	Olive farm	Wellington	33°34'21.0"S	19°'03'39.0"E	Imaging	n.a.
Argopistes sexvittatus	AM56	17-Jan-16	Wild	Ornamental tree	Stellenbosch	33°55'34.0"S	18°51'53.0"E	Imaging	n.a.
Argopistes sexvittatus	AM57	17-Jan-16	Wild	Ornamental tree	Stellenbosch	33°55'34.0"S	18°51'53.0"E	Imaging	n.a.
Argopistes sexvittatus	AM68	17-Jan-16	Wild	Ornamental tree	Stellenbosch	33°55'34.0"S	18°51'53.0"E	DNA barcode	**Upon acceptance**

Argopistes sexvittatus	AM69	23-Jan-16	Wild	Ornamental tree	Stellenbosch	33°55'34.0"S	18°51'53.0"E	DNA barcode	**Upon acceptance**
Argopistes sexvittatus	AM71	24-Jan-16	Cultivated	Ornamental tree	Stellenbosch	33°56'13.2"S	18°51'13.2"E	DNA barcode	**Upon acceptance**
Argopistes sexvittatus	AM72	24-Jan-16	Cultivated	Ornamental tree	Stellenbosch	33°56'13.2"S	18°51'13.2"E	DNA barcode	**Upon acceptance**
Argopistes sexvittatus	AM74	24-Jan-16	Cultivated	Ornamental tree	Stellenbosch	33°56'13.2"S	18°51'13.2"E	DNA barcode	**Upon acceptance**
Argopistes sexvittatus	AM75	24-Jan-16	Cultivated	Ornamental tree	Stellenbosch	33°56'13.2"S	18°51'13.2"E	DNA barcode	**Upon acceptance**
Argopistes sexvittatus	AM77	24-Jan-16	Cultivated	Ornamental tree	Stellenbosch	33°56'13.2"S	18°51'13.2"E	DNA barcode	**Upon acceptance**
Argopistes sexvittatus	AM78	24-Jan-16	Cultivated	Ornamental tree	Stellenbosch	33°56'13.2"S	18°51'13.2"E	DNA barcode	**Upon acceptance**
Argopistes sexvittatus	AM80	28-Jan-16	Cultivated	Ornamental tree	Stellenbosch	33°56'13.2"S	18°51'13.2"E	DNA barcode	**Upon acceptance**
Argopistes sexvittatus	AM82	28-Jan-16	Cultivated	Ornamental tree	Stellenbosch	33°56'13.2"S	18°51'13.2"E	DNA barcode	**Upon acceptance**
Argopistes sexvittatus	AM83	28-Jan-16	Cultivated	Ornamental tree	Stellenbosch	33°56'13.2"S	18°51'13.2"E	DNA barcode	**Upon acceptance**
Argopistes sexvittatus	AS001	13-Jan-16	Wild	Ornamental tree	Stellenbosch	33°55'34.0"S	18°51'53.0"E	DNA barcode	**Upon acceptance**
Argopistes sexvittatus	AS003	13-Jan-16	Wild	Ornamental tree	Stellenbosch	33°55'34.0"S	18°51'53.0"E	DNA barcode	**Upon acceptance**
Argopistes sexvittatus	AS005	13-Jan-16	Wild	Ornamental tree	Stellenbosch	33°55'34.0"S	18°51'53.0"E	DNA barcode	**Upon acceptance**
Argopistes sexvittatus	AS038	10-Nov-15	Cultivated	Olive farm	Wellington	33°34'21.0"S	19°'03'39.0"E	DNA barcode	**Upon acceptance**
Argopistes sexvittatus	AS078	04-Nov-15	Cultivated	Olive farm	Wellington	33°34'21.0"S	19°'03'39.0"E	DNA barcode	**Upon acceptance**
Argopistes sexvittatus	AS093	16-Jan-16	Cultivated	Ornamental tree	Stellenbosch	33°56'13.2"S	18°51'13.2"E	Imaging	n.a.
Argopistes sexvittatus	AS094	16-Jan-16	Cultivated	Ornamental tree	Stellenbosch	33°56'13.2"S	18°51'13.2"E	Imaging	n.a.
Argopistes sexvittatus	AS107	17-Jan-16	Wild	Ornamental tree	Stellenbosch	33°55'34.0"S	18°51'53.0"E	Imaging	n.a.
Argopistes sexvittatus	AS111	17-Jan-16	Wild	Ornamental tree	Stellenbosch	33°55'34.0"S	18°51'53.0"E	Imaging	n.a.
Argopistes sexvittatus	AS120	28-Jan-16	Cultivated	Ornamental tree	Stellenbosch	33°56'13.2"S	18°51'13.2"E	DNA barcode	**Upon acceptance**
