# Supplementary material for: Argopistes sexvittatus and Argopistes capensis (Chrysomelidae: Alticini): Mitogenomics and Phylogeny of Two Flea Beetles Affecting Olive Trees
Source: Genes (Basel). 2022 Nov 23;13(12):2195. doi: 10.3390/genes13122195 (PMC9777630; doi:10.3390/genes13122195)
Supplement: Supplementary file 1 [file genes-13-02195-s001.zip › Table S2 Mitogenomes phylogeny.pdf]

Table S2. List of the 64 mitogenomes used to assess the phylogenetic position of the olive flea beetles *Argopistes capensis* and *Argopistes sexvittatus* (Coleoptera: Chrysomelidae) in the tribe Alticini. *Aeolesthes oenochrous* (Cerambycinae), *Anoplophora glabripennis* (Lamiinae), *Crioceris duodecimpunctata* (Criocerinae) and *Phaedon tumidulus* (Chrysomelinae) were used as outgroups.

**Species**	**GenBank**	**Larval feeding habit**	**Host plant family**	**Group of genera**
Acrocrypta assamensis	MG021086	unknown	Cucurbitaceae, Araceae	*Acrocrypta* group
Aeolesthes oenochrous (Cerambycinae)	AB703463	external leaf feeder	various	Outgroup
Agasicles hygrophila	NC_028332	external leaf feeder	Amaranthaceae, Polygonaceae	*Disonycha* group
Altica ericeti	KX943460	external leaf feeder	Ericaceae	*Altica* group
Altica viridicyanea	NC_048472	external leaf feeder	Geraniaceae	*Altica* group
Anoplophora glabripennis (Lamiinae)	NC_008221	external leaf feeder	various	Outgroup
Aphthona lutescens	KX943361	leaf miner	Lythraceae	*Longitarsus group*
Aphthona strigosa	MF979907	leaf miner	Euphorbiaceae	*Longitarsus group*
Apteropeda orbiculata	KX943507	leaf miner	Plantaginaceae	*Sphaeroderma* group
Apteropeda ovulum	KX943422	leaf miner	Plantaginaceae	*Sphaeroderma* group
Argopistes capensis AC3	UPON ACCEPTANCE	leaf miner	Oleaceae	*Sphaeroderma* group
Argopistes sexvittatus AG01	UPON ACCEPTANCE	leaf miner	Oleaceae	*Sphaeroderma* group
Argopistes sexvittatus AG08	UPON ACCEPTANCE	leaf miner	Oleaceae	*Sphaeroderma* group
Argopistes sp. BMNH 846608	MF960114	leaf miner	Oleaceae	*Sphaeroderma* group
Argopistes tsekooni	NC_045929	leaf miner	Oleaceae	*Sphaeroderma* group
Asphaera sp. REN-2018	MF351886	external leaf feeder	Lamiaceae, Loganiaceae, Verbenaceae	*Oedionychis* group

Batophila aerata	KX943466	(?) root feeder	Rosaceae	Unclear
Bikasha collaris	MF960113	root feeder	Euphorbiaceae	*Aphthona* group
Blepharida rhois	MF351884	External leaf feeder	Anacardiaceae	*Blepharida* group
Chabria nr. angulicollis REN-2018	MF351887	Unknown	Melastomataceae	*Chabria* group
Chaetocnema arida	KX943445	Leaf miner	Poaceae	*Chaetocnema* group
Chaetocnema pelagica	NC_041170	Leaf miner	Juncaceae	*Chaetocnema* group
Chaetocnema tibialis	KX943476	Leaf miner	Chenopodiaceae	*Chaetocnema* group
Crepidodera pluta	KX087265	(?) Root feeder	Salicaceae	Unclear
Crioceris duodecimpunctata (Criocerinae)	NC_003372	External leaf feeder	Cucurbitaceae, Asparagaceae	Outgroup
Diphaltica sp. REN-2018	MF351888	(?) External leaf feeder	Aquipholiaceae	*Oedionychis* group
Epitrix abeillei	MF960107	Root feeder	Solanaceae	*Chaetocnema* group
Halticorcus sp. BMNH 846646	MF979902	Leaf miner	Oleandraceae, Polypodiaceae, Aspidiaceae	unclear
Hemipyxis plagioderoides	MF960118	External leaf feeder	Lamiaceae, Plantaginaceae	*Oedionychis* group
Hespera cyanea	MF960119	(?) External leaf feeder	various	Unclear
Hespera obesula	MF960120	(?) External leaf feeder	various	Unclear
Hyphasis sp. REN-2018	MG021088	External leaf feeder	Verbenaceae, Lamiaceae	*Oedionychis* group
Lactica sp. BMNH 846625	MF960122	(?) Root feeder	various	Unclear
Lanka ramakrishnai	MF960108	(?) Root feeder	Piperaceae	*Lanka* group
Laotzeus sp. BMNH 846624	MF960121	Unknown	Unknown	Unclear
Lipromima minuta	MF960123	Unknown	Anacardiaceae	*Chaetocnema* group
Longitarsus aeneus	KX943357	Root feeder	Boraginaceae	*Longitarsus* group
Longitarsus atricillus	KX943363	Root feeder	Boraginaceae	*Longitarsus* group
Luperomorpha hainana	MF960124	(?) Root feeder	Unknown	Unclear
Lypnea pubipennis	MF960110	Unknown	Flacourtiaceae, Passifloraceae	Unclear

Macrohaltica subplicata	NC_041169	External leaf feeder	Gunneraceae	*Altica* group
Mandarella flaviventris	MF979905	Unknown	Unknown	Unclear
Mandarella nipponensis	MF979904	Unknown	Unknown	Unclear
Mantura chrysanthemi	KX943486	Leaf miner	Polygonaceae	Unknown
Neocrepidodera brevicollis	KX943440	(?) Leaf miner	Poaceae	*Chabria* group
Neocrepidodera transversa	MF351885	(?) Root feeder	Asteraceae	*Chabria* group
Nisotra sp. BMNH 846636	MF960109	Endophytes [(?) Root feeder]	various	*Nisotra* group
Nonarthra nigricolle	MF979894	External leaf feeder	various	*Nonarthra* group
Nonarthra nigripenne	MF979895	External leaf feeder	various	*Nonarthra* group
Novofoudrasia regularis	MF960117	(?) Leaf miner	Fabaceae	Unknown
Oedionychus cinctus	MF979909	External leaf feeder	Plantaginaceae	*Oedionychis* group
Omophoita sp. REN-2018	MG021084	External leaf feeder	Lamiaceae, Verbenaceae, Boraginaceae	*Oedionychis group*
Orestia punctipennis	KX943441	Unknown	Lamiaceae	Unknown
Phaedon tumidulus (Chrysomelinae)	KX943499	External leaf feeder	Apiaceae	Outgroup
Philopona vibex	MF979898	External leaf feeder	Plantaginaceae, Verbenaceae, Acanthaceae	*Oedionychis group*
Phygasia gracilicornis	MF979896	(?) Root feeder	unknown	*Phygasia* group
Phygasia ornata	MF979897	(?) Root feeder	Asclepiadaceae	*Phygasia* group
Phyllotreta cruciferae	KX943506	Leaf miner	Brassicaceae	(?) *Aphthona* group
Phyllotreta foudrasi	KX943502	Leaf miner	Brassicaceae	(?) *Aphthona* group
Podagrica sp. BMNH 848524	MF979910	root feeder	Malvaceae	*Nisotra* group
Podontia lutea	MF979899	external leaf feeder	Anacardiaceae	*Blepharida* group
Psylliodes affinis	KX943355	leaf miner	Solanaceae	*Chaetocnema* group
Psylliodes chrysocephala	KX943483	leaf miner	Brassicaceae	*Chaetocnema* group
Sangariola fortunei	MF979901	(?) root feeder	Smilacaceae, Liliaceae, Araceae	Unclear
Sinocrepis fulva	MF960111	(?) root feeder	unknown	*Nisotra* group
Syphrea sp. REN-2018	MG021085	external leaf feeder	Euphorbiaceae	*Altica* group

Tegyrius keralaensis	MF960112	(?) root feeder	Piperaceae	*Lanka* group
Xuthea yunnanensis	MF979906	(?) leaf miner	Scrophulariaceae	*Chaetocnema* group
