# Supplementary material for: Argopistes sexvittatus and Argopistes capensis (Chrysomelidae: Alticini): Mitogenomics and Phylogeny of Two Flea Beetles Affecting Olive Trees
Source: Genes (Basel). 2022 Nov 23;13(12):2195. doi: 10.3390/genes13122195 (PMC9777630; doi:10.3390/genes13122195)
Supplement: Supplementary file 1 [file genes-13-02195-s001.zip › Table S3 Mitogenome features.pdf]

**Table S3.** Main features of the complete mitochondrial genome of the olive flea beetles *Argopistes capensis* and *Argopistes sexvittatus* (Coleoptera: Chrysomelidae). J – majority strand; N – minority strand; AC – Anticodon; IGN – number of intergenic nucleotides (negative values indicate overlapping between genes).

| | | | | ***Argopistes capensis* AC3** | | | | | ***Argopistes sexvittatus* AG01 (striped morphotype)** | | | | | ***Argopistes sexvittatus* AG08 (black morphotype)** | | | | |
|---|---|---|---|---|---|---|---|---|---|---|---|---|---|---|---|---|---|---|
| **Gene** | **Code** | **Strand** | **AC** | **Coordinates** | **bp** | **Start** | **Stop** | **IGN** | **Coordinates** | **bp** | **Start** | **Stop** | **IGN** | **Coordinates** | **bp** | **Start** | **Stop** | **IGN** |
| tRNA$^{Ile}$ | I | J | GAT | 2-63 | 61 | - | - | - | 1-63 | 63 | - | - | - | 1-63 | 63 | - | - | - |
| tRNA$^{Gln}$ | Q | N | TTG | 65-135 | 71 | - | - | 0 | 75-145 | 71 | - | - | 11 | 75-145 | 71 | - | - | 11 |
| tRNA$^{Met}$ | M | J | CAT | 134-202 | 69 | - | - | -1 | 145-213 | 69 | - | - | -1 | 145-213 | 69 | - | - | -1 |
| ND2 | - | J | - | 203-1214 | 1012 | ATT | T-- | 0 | 214-1225 | 1012 | ATT | T-- | 0 | 214-1225 | 1012 | ATT | T-- | 0 |
| tRNA$^{Trp}$ | W | J | TCA | 1214-1279 | 66 | - | - | -1 | 1225-1290 | 66 | - | - | -1 | 1225-1290 | 66 | - | - | -1 |
| tRNA$^{Cys}$ | C | N | GCA | 1270-1335 | 66 | - | - | -10 | 1281-1346 | 66 | - | - | -10 | 1281-1346 | 66 | - | - | -10 |
| tRNA$^{Tyr}$ | Y | N | GTA | 1336-1399 | 64 | - | - | 0 | 1347-1410 | 64 | - | - | 0 | 1346-1410 | 65 | - | - | -1 |
| COI | - | J | - | 1401-2934 | 1534 | AAT | T-- | 1 | 1412-2945 | 1534 | AAT | T-- | 1 | 1412-2945 | 1534 | AAT | T-- | 1 |
| tRNA$^{Leu2}$ | L2 | J | TAA | 2934-3002 | 69 | - | - | -1 | 2945-3013 | 69 | - | - | -1 | 2945-3013 | 69 | - | - | -1 |
| COII | - | J | - | 3002-3689 | 688 | ATG | T-- | -1 | 3013-3700 | 668 | ATG | T-- | -1 | 3013-3700 | 688 | ATG | T-- | -1 |
| tRNA$^{Lys}$ | K | J | TTT | 3690-3761 | 72 | - | - | 0 | 3701-3772 | 72 | - | - | 0 | 3701-3772 | 72 | - | - | 0 |
| tRNA$^{Asp}$ | D | J | GTC | 3760-3827 | 68 | - | - | -2 | 3771-3835 | 65 | - | - | -2 | 3771-3836 | 66 | - | - | -2 |
| ATP8 | - | J | - | 3828-3983 | 156 | ATT | TAA | 0 | 3836-3991 | 156 | ATC | TAA | 0 | 3837-3992 | 156 | ATC | TAA | 0 |
| ATP6 | - | J | - | 3977-4648 | 672 | ATG | TAA | -7 | 3985-4653 | 669 | ATG | TAA | -7 | 3986-4654 | 669 | ATG | TAA | -7 |
| COIII | - | J | - | 4648-5429 | 782 | ATG | TA- | -1 | 4653-5434 | 782 | ATG | TA- | -1 | 4654-5435 | 782 | ATG | TA- | -1 |
| tRNA$^{Gly}$ | G | J | TCC | 5430-5493 | 64 | - | - | 0 | 5435-5499 | 65 | - | - | 0 | 5436-5499 | 64 | - | - | 0 |
| ND3 | - | J | - | 5494-5845 | 352 | ATT | T-- | -1 | 5502-5835 | 352 | ATT | T-- | 0 | 5500-5851 | 352 | ATT | T-- | 0 |
| tRNA$^{Ala}$ | A | J | TGC | 5845-5911 | 67 | - | - | -1 | 5851-5916 | 66 | - | - | -1 | 5851-5916 | 66 | - | - | -1 |
| tRNA$^{Arg}$ | R | J | TCG | 5910-5972 | 63 | - | - | -2 | 5915-5979 | 65 | - | - | -2 | 5915-5979 | 65 | - | - | -2 |
| tRNA$^{Asn}$ | N | J | GTT | 5972-6037 | 66 | - | - | -1 | 5979-6043 | 65 | - | - | -1 | 5979-6043 | 65 | - | - | -1 |
| tRNA$^{Ser1}$ | S1 | J | TCT | 6038-6096 | 59 | - | - | 0 | 6044 -6101 | 58 | - | - | 0 | 6044-6101 | 58 | - | - | 0 |
| tRNA$^{Glu}$ | E | J | TTC | 6096-6161 | 66 | - | - | -1 | 6101-6165 | 66 | - | - | -1 | 6101-6166 | 66 | - | - | -1 |

| | | | | | | | | | | | | | | | | | | |
|---|---|---|---|---|---|---|---|---|---|---|---|---|---|---|---|---|---|---|
| tRNA$^{Phe}$ | F | N | GAA | 6157-6221 | 65 | - | - | -5 | 6162-6226 | 65 | - | - | -4 | 6162-6226 | 65 | - | - | -5 |
| ND5 | - | N | - | 6221-7919 | 1699 | ATT | T-- | -1 | 6226-7924 | 1699 | ATT | T-- | -1 | 6226-7924 | 1699 | ATT | T-- | -1 |
| tRNA$^{His}$ | H | N | GTG | 7920-7981 | 62 | - | - | 0 | 7925-7986 | 62 | - | - | 0 | 7925-7986 | 62 | - | - | 0 |
| ND4 | - | N | - | 7981-9298 | 1318 | ATG | T-- | -1 | 7987-9304 | 1318 | ATG | T-- | -1 | 7987-9304 | 1318 | ATG | T-- | 0 |
| ND4L | - | N | - | 9292-9573 | 282 | ATG | TAA | -7 | 9298-9579 | 282 | ATG | TAA | -7 | 9298-9579 | 282 | ATG | TAA | -7 |
| tRNA$^{Thr}$ | T | J | TGT | 9577-9643 | 67 | - | - | 3 | 9583-9649 | 67 | - | - | 3 | 9583-9649 | 67 | - | - | 3 |
| tRNA$^{Pro}$ | P | N | TGG | 9641-9706 | 66 | - | - | -3 | 9647-9712 | 66 | - | - | -3 | 9647-9712 | 66 | - | - | -3 |
| ND6 | - | J | - | 9708 - 10205 | 498 | ATT | TAA | 1 | 9714 - 10211 | 498 | ATT | TAA | 1 | 9714 -10211 | 498 | ATT | TAA | 1 |
| CYTB | - | J | - | 10205 - 11342 | 1138 | ATG | T-- | -1 | 10211-11348 | 1138 | ATG | T-- | -1 | 10211 -11348 | 1138 | ATG | T-- | -1 |
| tRNA$^{Ser2}$ | S2 | J | TGA | 11343 - 11409 | 67 | - | - | 0 | 11349 - 11415 | 67 | - | - | 0 | 11349 -11415 | 67 | - | - | 0 |
| ND1 | - | N | - | 11427- 12377 | 951 | TTG | TAG | 17 | 11433-12383 | 951 | TTG | TAG | 17 | 11433-12383 | 951 | TTG | TAG | 17 |
| tRNA$^{Leu1}$ | L1 | N | TAG | 12443- 12378 | 66 | - | - | 0 | 12384-12449 | 66 | - | - | 0 | 12449-12384 | 66 | - | - | 0 |
| 16s rRNA | - | N | - | 12444- 13721 | 1278 | - | - | 0 | 12450-13727 | 1278 | - | - | 0 | 12450-13728 | 1279 | - | - | 0 |
| tRNA$^{Val}$ | - | N | TAC | 13722- 13790 | 69 | - | - | 0 | 13728-13795 | 68 | - | - | 0 | 13729-13796 | 68 | - | - | 0 |
| 12s rRNA | - | N | - | 13791- 14528 | 738 | - | - | 0 | 13796-14533 | 738 | - | - | 0 | 13797-14534 | 738 | - | - | 0 |
| AT-rich region | - | - | - | 14529 - 16543 | 2015 | - | - | 0 | 14534-16542 | 2009 | - | - | 0 | 14535 -16566 | 2032 | - | - | 0 |
