# Supplementary material for: Argopistes sexvittatus and Argopistes capensis (Chrysomelidae: Alticini): Mitogenomics and Phylogeny of Two Flea Beetles Affecting Olive Trees
Source: Genes (Basel). 2022 Nov 23;13(12):2195. doi: 10.3390/genes13122195 (PMC9777630; doi:10.3390/genes13122195)
Supplement: Supplementary file 1 [file genes-13-02195-s001.zip › Table S4 Nucleotide composition.pdf]

**Table S4.** Nucleotide compositions of the complete mitochondrial sequences of the olive flea beetles *Argopistes capensis* and *Argopistes sexvittatus* (Coleoptera: Chrysomelidae: Alticini). AT-skew = (A - T)/(A + T); CG-skew = (G - C)/(G + C).

| | ***Argopistes capensis* AC3** | | | | | | | | | |
|---|---|---|---|---|---|---|---|---|---|---|
| **Gene/region** | **A%** | **C%** | **G%** | **T%** | **A+T%** | **G+C%** | **AT-skew** | **GC-skew** | **Size (bp)** | **% (size)** |
| **COI** | 33.0 | 14.3 | 14.1 | 38.6 | 71.6 | 28.4 | -0.1 | 0.0 | 1534 | 9.3 |
| **COII** | 37.1 | 13.4 | 9.6 | 40.0 | 77.1 | 23.0 | 0.0 | -0.2 | 688 | 4.2 |
| **COIII** | 33.4 | 13.8 | 11.8 | 40.2 | 73.6 | 25.6 | -0.1 | -0.1 | 782 | 4.7 |
| **CYTB** | 35.9 | 13.2 | 10.9 | 40.0 | 75.9 | 24.1 | -0.1 | -0.1 | 1138 | 6.9 |
| **ATP6** | 37.9 | 13.7 | 8.8 | 39.6 | 77.5 | 22.5 | 0.0 | -0.2 | 672 | 4.1 |
| **ATP8** | 47.4 | 7.7 | 1.9 | 42.9 | 90.3 | 9.6 | 0.0 | -0.6 | 156 | 0.9 |
| **ND1** | 49.0 | 13.7 | 7.8 | 29.5 | 78.5 | 21.5 | 0.2 | -0.3 | 951 | 5.7 |
| **ND2** | 39.1 | 12.0 | 6.5 | 42.4 | 81.5 | 18.5 | 0.0 | -0.3 | 1012 | 6.1 |
| **ND3** | 36.6 | 13.4 | 7.4 | 42.6 | 79.2 | 20.8 | -0.1 | -0.3 | 352 | 2.1 |
| **ND4** | 51.1 | 12.3 | 7.2 | 29.4 | 80.5 | 19.5 | 0.3 | -0.3 | 1318 | 8.0 |
| **ND4L** | 53.2 | 11.7 | 4.6 | 30.5 | 83.7 | 16.3 | 0.3 | -0.4 | 282 | 1.7 |
| **ND5** | 48.1 | 11.8 | 8.0 | 32.0 | 80.1 | 19.8 | 0.2 | -0.2 | 1699 | 10.3 |
| **ND6** | 39.8 | 10.4 | 5.2 | 44.6 | 84.4 | 15.6 | -0.1 | -0.3 | 498 | 3.0 |
| **16s rRNA** | 44.6 | 11.4 | 5.6 | 38.3 | 82.9 | 17.0 | 0.1 | -0.3 | 1278 | 7.7 |
| **12s rRNA** | 44.2 | 11.2 | 5.8 | 38.8 | 83.0 | 17.0 | 0.1 | -0.3 | 738 | 4.5 |
| **Total PCGs** | 41.5 | 12.8 | 9.0 | 36.7 | 78.2 | 21.8 | 0.1 | -0.2 | 11082 | 67.0 |
| **Total tRNAs** | 41.6 | 11.3 | 8.5 | 38.6 | 80.2 | 19.8 | 0.0 | -0.1 | 1383 | 8.4 |
| **Total rRNAs** | 44.4 | 11.4 | 5.7 | 38.5 | 82.9 | 17.1 | 0.1 | -0.3 | 2016 | 12.2 |
| **AT-rich region** | 49.2 | 4.0 | 5.4 | 41.3 | 90.5 | 9.4 | 0.1 | 0.1 | 2015 | 12.2 |
| **Complete mtDNA** | 42.5 | 11.8 | 8.2 | 37.5 | 80 | 20.0 | 0.06 | -0.18 | 16543 | 100.0 |

| | ***Argopistes sexvittatus* AG01 (striped morphotype)** | | | | | | | | | |
|---|---|---|---|---|---|---|---|---|---|---|
| **Gene/region** | **A%** | **C%** | **G%** | **T%** | **A+T%** | **G+C%** | **AT-skew** | **GC-skew** | **Size (bp)** | **% (size)** |
| **COI** | 32.7 | 14.4 | 14.0 | 38.9 | 71.6 | 28.4 | -0.1 | 0.0 | 1534 | 9.3 |
| **COII** | 38.1 | 14.8 | 9.6 | 37.5 | 75.6 | 24.4 | 0.0 | -0.2 | 688 | 0.0 |
| **COIII** | 34.0 | 14.1 | 11.8 | 40.2 | 74.2 | 25.9 | -0.1 | -0.1 | 782 | 4.7 |
| **CYTB** | 35.7 | 12.5 | 10.6 | 41.2 | 76.9 | 23.1 | -0.1 | -0.1 | 1138 | 6.9 |
| **ATP6** | 37.3 | 13.2 | 9.6 | 40.0 | 77.3 | 22.8 | 0.0 | -0.2 | 669 | 4.0 |
| **ATP8** | 44.8 | 10.4 | 2.6 | 42.2 | 87.0 | 13.0 | 0.0 | -0.6 | 156 | 0.9 |
| **ND1** | 49.3 | 11.9 | 8.0 | 30.8 | 80.1 | 19.9 | 0.2 | -0.2 | 951 | 5.7 |
| **ND2** | 39.1 | 11.7 | 6.8 | 42.4 | 81.5 | 18.5 | 0.0 | -0.3 | 1012 | 6.1 |
| **ND3** | 37.5 | 11.1 | 8.2 | 43.2 | 80.7 | 19.3 | -0.1 | -0.2 | 352 | 2.1 |
| **ND4** | 51.3 | 12.1 | 7.6 | 29.1 | 80.4 | 19.7 | 0.3 | -0.2 | 1318 | 8.0 |
| **ND4L** | 53.9 | 10.3 | 4.3 | 31.6 | 85.5 | 14.6 | 0.3 | -0.4 | 282 | 1.7 |
| **ND5** | 49.4 | 11.5 | 7.8 | 31.3 | 80.7 | 19.3 | 0.2 | -0.2 | 1699 | 10.3 |
| **ND6** | 40.4 | 8.0 | 5.2 | 46.3 | 86.7 | 13.2 | -0.1 | -0.2 | 497 | 3.0 |
| **16s rRNA** | 44.6 | 11.6 | 5.6 | 38.4 | 83.0 | 17.2 | 0.1 | -0.3 | 1278 | 7.7 |
| **12s rRNA** | 43.2 | 11.5 | 6.0 | 39.3 | 82.5 | 17.5 | 0.0 | -0.3 | 738 | 4.5 |
| **Total PCGs** | 41.7 | 12.4 | 9.1 | 36.8 | 78.5 | 21.5 | 0.1 | -0.2 | 11078 | 67.0 |
| **Total tRNAs** | 41.8 | 11.0 | 8.2 | 39.0 | 80.8 | 19.2 | 0.0 | -0.1 | 1382 | 8.4 |
| **Total rRNAs** | 44.1 | 11.4 | 5.6 | 38.7 | 82.8 | 17.0 | 0.1 | -0.3 | 2016 | 12.2 |

| | | | | | | | | | | |
|---|---|---|---|---|---|---|---|---|---|---|
| **AT-rich region** | 46.1 | 8.2 | 6.1 | 39.6 | 85.7 | 14.3 | 0.1 | -0.1 | 2009 | 12.1 |
| **Complete mtDNA** | 42.4 | 11.7 | 8.3 | 37.5 | 79.9 | 20.0 | 0.1 | -0.2 | 16542 | 100.0 |

| | ***Argopistes sexvittatus* AG08 (black morphotype)** | | | | | | | | | |
|---|---|---|---|---|---|---|---|---|---|---|
| **Gene/region** | **A%** | **C%** | **G%** | **T%** | **A+T%** | **G+C%** | **AT-skew** | **GC-skew** | **Size (bp)** | **% (size)** |
| **COI** | 32.7 | 14.4 | 14.0 | 38.9 | 71.6 | 28.4 | -0.1 | 0.0 | 1534 | 9.3 |
| **COII** | 37.9 | 14.8 | 9.7 | 37.5 | 75.4 | 24.5 | 0.0 | -0.2 | 688 | 4.2 |
| **COIII** | 33.9 | 14.1 | 11.8 | 40.3 | 74.2 | 25.9 | -0.1 | -0.1 | 782 | 4.7 |
| **CYTB** | 35.6 | 12.7 | 10.6 | 41.0 | 76.6 | 23.3 | -0.1 | -0.1 | 1138 | 6.9 |
| **ATP6** | 37.4 | 13.0 | 9.6 | 40.1 | 77.5 | 22.6 | 0.0 | -0.2 | 669 | 4.0 |
| **ATP8** | 45.0 | 9.7 | 2.6 | 42.4 | 87.4 | 12.3 | 0.0 | -0.6 | 156 | 0.9 |
| **ND1** | 49.3 | 11.9 | 8.0 | 30.8 | 80.1 | 19.9 | 0.2 | -0.2 | 951 | 5.7 |
| **ND2** | 38.9 | 11.7 | 7.0 | 42.4 | 81.3 | 18.7 | 0.0 | -0.3 | 1012 | 6.1 |
| **ND3** | 37.5 | 11.1 | 8.2 | 43.2 | 80.7 | 19.3 | -0.1 | -0.2 | 352 | 2.1 |
| **ND4** | 51.3 | 12.1 | 7.6 | 29.1 | 80.4 | 19.7 | 0.3 | -0.2 | 1318 | 8.0 |
| **ND4L** | 53.9 | 10.3 | 4.3 | 31.6 | 85.5 | 14.6 | 0.3 | -0.4 | 282 | 1.7 |
| **ND5** | 49.6 | 11.5 | 7.7 | 31.3 | 80.9 | 19.2 | 0.2 | -0.2 | 1699 | 10.3 |
| **ND6** | 40.4 | 8.0 | 5.2 | 46.3 | 86.7 | 13.2 | -0.1 | -0.2 | 498 | 3.0 |
| **16s rRNA** | 44.6 | 11.3 | 5.6 | 38.4 | 83.0 | 16.9 | 0.1 | -0.3 | 1279 | 7.7 |
| **12s rRNA** | 43.2 | 11.5 | 6.0 | 39.3 | 82.5 | 17.5 | 0.0 | -0.3 | 738 | 4.5 |
| **Total PCGs** | 41.7 | 12.4 | 9.1 | 36.8 | 78.5 | 21.5 | 0.1 | -0.2 | 11079 | 66.9 |
| **Total tRNAs** | 41.8 | 10.9 | 8.1 | 39.2 | 81.0 | 19.0 | 0.0 | -0.1 | 1452 | 8.8 |
| **Total rRNAs** | 44.1 | 11.4 | 5.8 | 38.7 | 82.8 | 17.2 | 0.1 | -0.3 | 2017 | 12.2 |
| **AT-rich region** | 45.8 | 7.5 | 5.6 | 41.1 | 86.9 | 13.1 | 0.1 | -0.1 | 2032 | 12.3 |
| **Complete mtDNA** | 42.4 | 11.6 | 8.2 | 37.7 | 80.1 | 19.8 | 0.1 | -0.2 | 16566 | 100.0 |
