# Supplementary material for: Argopistes sexvittatus and Argopistes capensis (Chrysomelidae: Alticini): Mitogenomics and Phylogeny of Two Flea Beetles Affecting Olive Trees
Source: Genes (Basel). 2022 Nov 23;13(12):2195. doi: 10.3390/genes13122195 (PMC9777630; doi:10.3390/genes13122195)
Supplement: Supplementary file 1 [file genes-13-02195-s001.zip › Table S5 Codon usage.pdf]

Table S5. Codon usage in the complete mitochondrial genomes of the olive flea beetles *Argopistes capensis* and *Argopistes sexvittatus* (Coleoptera: Chrysomelidae). AA - Amino acids are labelled according to the IUPAC-IUB single-letter codes. N - the total number of occurrences in all protein-coding genes, RSCU - relative synonymous codon usage.

***Argopistes capensis* AC3**								***Argopistes sexvittatus* AG01 (striped morphotype)**								***Argopistes sexvittatus* AG08 (black morphotype)**							
**AA**	**Codon**	**N**	**RSCU**	**AA**	**Codon**	**N**	**RSCU**	**AA**	**Codon**	**N**	**RSCU**	**AA**	**Codon**	**N**	**RSCU**	**AA**	**Codon**	**N**	**RSCU**	**AA**	**Codon**	**N**	**RSCU**
F	UUU	352	1.56	Y	UAU	252	1.54	F	UUU	331	1.57	Y	UAU	269	1.58	F	UUU	344	1.55	Y	UAU	301	1.64
	UUC	99	0.44		UAC	75	0.46		UUC	91	0.43		UAC	72	0.42		UUC	100	0.45		UAC	67	0.36
L	UUA	304	2.95	H	CAU	80	1.58	L	UUA	327	3.30	H	CAU	76	1.60	L	UUA	363	3.47	H	CAU	80	1.65
	UUG	64	0.62		CAC	21	0.42		UUG	57	0.58		CAC	19	0.4		UUG	48	0.46		CAC	17	0.35
	CUU	101	0.98	Q	CAA	87	1.47		CUU	79	0.80	Q	CAA	105	1.72		CUU	86	0.82	Q	CAA	84	1.57
	CUC	25	0.24		CAG	31	0.53		CUC	31	0.31		CAG	17	0.28		CUC	22	0.21		CAG	23	0.43
	CUA	88	0.85	N	AAU	428	1.62		CUA	75	0.76	N	AAU	403	1.67		CUA	93	0.89	N	AAU	396	1.54
	CUG	36	0.35		AAC	99	0.38		CUG	25	0.25		AAC	81	0.33		CUG	15	0.14		AAC	119	0.46
I	AUU	380	1.65	K	AAA	501	1.76	I	AUU	415	1.62	K	AAA	431	1.75	I	AUU	398	1.62	K	AAA	453	1.63
	AUC	80	0.35		AAG	69	0.24		AUC	96	0.38		AAG	62	0.25		AUC	92	0.38		AAG	104	0.37
M	AUA	302	1.72	D	GAU	64	1.51	M	AUA	329	1.77	D	GAU	63	1.54	M	AUA	321	1.72	D	GAU	50	1.43
	AUG	50	0.28		GAC	21	0.49		AUG	43	0.23		GAC	19	0.46		AUG	53	0.28		GAC	20	0.57
V	GUU	36	1.48	E	GAA	109	1.63	V	GUU	42	1.49	E	GAA	67	1.79	V	GUU	31	1.57	E	GAA	84	1.62
	GUC	13	0.54		GAG	25	0.37		GUC	9	0.32		GAG	8	0.21		GUC	9	0.46		GAG	20	0.38
	GUA	36	1.48	C	UGU	26	0.95		GUA	54	1.91	C	UGU	23	1.10		GUA	34	1.72	C	UGU	33	0.97
	GUG	12	0.49		UGC	29	1.05		GUG	8	0.28		UGC	19	0.90		GUG	5	0.25		UGC	35	1.03
S	UCU	72	1.66	W	UGA	62	1.48	S	UCU	74	1.33	W	UGA	77	1.50	S	UCU	73	1.35	W	UGA	71	1.22
	UCC	32	0.74		UGG	22	0.52		UCC	38	0.68		UGG	26	0.50		UCC	49	0.91		UGG	45	0.78
	UCA	82	1.89	R	CGU	7	0.93		UCA	100	1.80	R	CGU	6	0.83		UCA	99	1.83	R	CGU	10	0.89
	UCG	14	0.32		CGC	3	0.40		UCG	14	0.25		CGC	0	0.00		UCG	14	0.26		CGC	5	0.44
P	CCU	51	1.51		CGA	19	2.53	P	CCU	42	1.30		CGA	20	2.76	P	CCU	30	1.05		CGA	24	2.13
	CCC	36	1.07		CGG	1	0.13		CCC	33	1.02		CGG	3	0.41		CCC	29	1.02		CGG	6	0.53

	CCA	41	1.21	S	AGU	34	0.78		CCA	49	1.52	S	AGU	35	0.63		CCA	50	1.75	S	AGU	45	0.83
	CCG	7	0.21		AGC	24	0.55		CCG	5	0.16		AGC	53	0.95		CCG	5	0.18		AGC	44	0.81
T	ACU	70	1.48		AGA	53	1.22	T	ACU	76	1.34		AGA	102	1.84	T	ACU	74	1.44		AGA	63	1.17
	ACC	44	0.93		AGG	37	0.85		ACC	39	0.69		AGG	28	0.50		ACC	40	0.78		AGG	45	0.83
	ACA	64	1.35	G	GGU	23	1.35		ACA	93	1.64	G	GGU	25	1.04		ACA	80	1.55	G	GGU	17	1.05
	ACG	11	0.23		GGC	9	0.53		ACG	19	0.33		GGC	5	0.21		ACG	12	0.23		GGC	8	0.49
A	GCU	27	1.48		GGA	26	1.53	A	GCU	40	1.82		GGA	57	2.38	A	GCU	25	1.47		GGA	36	2.22
	GCC	16	0.88		GGG	10	0.59		GCC	17	0.77		GGG	9	0.38		GCC	14	0.82		GGG	4	0.25
	GCA	28	1.53						GCA	30	1.36						GCA	26	1.53				
	GCG	2	0.11						GCG	1	0.05						GCG	3	0.18				
